# Three Blind Moles: Molecular Evolutionary Insights on the Tempo and Mode of Convergent Eye Degeneration in *Notoryctes typhlops* (Southern Marsupial Mole) and Two Chrysochlorids (Golden Moles)

**DOI:** 10.3390/genes14112018

**Published:** 2023-10-28

**Authors:** Mark S. Springer, Christopher A. Emerling, John Gatesy

**Affiliations:** 1 Department of Evolution, Ecology, and Organismal Biology, University of California, Riverside, CA 92521, USA; 2 Biology Department, Reedley College, Reedley, CA 93654, USA; christopher.emerling@reedleycollege.edu; 3 Division of Vertebrate Zoology, American Museum of Natural History, New York, NY 10024, USA; johngatesy2@gmail.com

**Keywords:** Chrysochloridae, crystallin, *Notoryctes*, phototransduction, pseudogene

## Abstract

Golden moles (Chrysochloridae) and marsupial moles (Notoryctidae) are textbook examples of convergent evolution. Both taxa are highly adapted to subterranean lifestyles and have powerful limbs for digging through the soil/sand, ears that are adapted for low-frequency hearing, vestigial eyes that are covered by skin and fur, and the absence of optic nerve connections between the eyes and the brain. The eyes of marsupial moles also lack a lens as well as retinal rods and cones. Two hypotheses have been proposed to account for the greater degeneracy of the eyes of marsupial moles than golden moles. First, marsupial moles may have had more time to adapt to their underground habitat than other moles. Second, the eyes of marsupial moles may have been rapidly and recently vestigialized to (1) reduce the injurious effects of sand getting into the eyes and (2) accommodate the enlargement of lacrimal glands that keep the nasal cavity moist and prevent the entry of sand into the nasal passages during burrowing. Here, we employ molecular evolutionary methods on DNA sequences for 38 eye genes, most of which are eye-specific, to investigate the timing of relaxed selection (=neutral evolution) for different groups of eye-specific genes that serve as proxies for distinct functional components of the eye (rod phototransduction, cone phototransduction, lens/cornea). Our taxon sampling included 12 afrothere species, of which two are golden moles (*Amblysomus hottentotus, Chrysochloris asiatica*), and 28 marsupial species including two individuals of the southern marsupial mole (*Notoryctes typhlops*). Most of the sequences were mined from databases, but we also provide new genome data for *A. hottentotus* and one of the two *N. typhlops* individuals. Even though the eyes of golden moles are less degenerate than the eyes of marsupial moles, there are more inactivating mutations (e.g., frameshift indels, premature stop codons) in their cone phototransduction and lens/cornea genes than in orthologous genes of the marsupial mole. We estimate that cone phototransduction recovery genes were inactivated first in each group, followed by lens/cornea genes and then cone phototransduction activation genes. All three groups of genes were inactivated earlier in golden moles than in marsupial moles. For the latter, we estimate that lens/cornea genes were inactivated ~17.8 million years ago (MYA) when stem notoryctids were burrowing in the soft soils of Australian rainforests. Selection on phototransduction activation genes was relaxed much later (5.38 MYA), during the early stages of Australia’s aridification that produced coastal sand plains and eventually sand dunes. Unlike cone phototransduction activation genes, rod phototransduction activation genes are intact in both golden moles and one of the two individuals of *N. typhlops*. A second marsupial mole individual has just a single inactivating mutation in one of the rod phototransduction activation genes (*PDE6B*). One explanation for this result is that some rod phototransduction activation genes are pleiotropic and are expressed in extraocular tissues, possibly in conjunction with sperm thermotaxis.

## 1. Introduction

**Figure 1:**
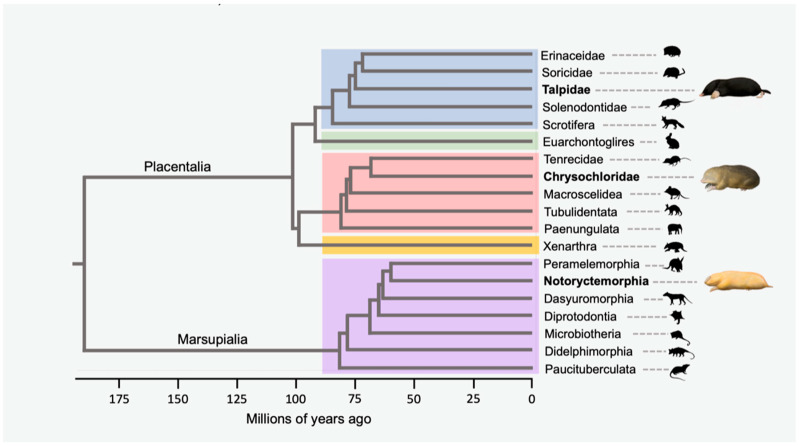
Simplified phylogeny for therian mammals that shows the placement of three mole families (Chrysochloridae, Talpidae, Notoryctidae). Divergence times are from Meredith et al. [11]. Shaded boxes are as follows: blue = Laurasiatheria; green = Euarchontoglires; red = Afrotheria; orange = Xenarthra; and purple = Marsupialia. Silhouettes are for the same species or a closely related species.

Convergent evolution is a central theme in the history of life on Earth [1,2], and the products of convergence comprise a natural laboratory, complete with replicated experiments, for elucidating regions of the genome that underlie specific phenotypic traits [3,4,5]. Numerous definitions of convergent evolution have been proposed, but at their core, most center on the independent evolution of similar traits in multiple lineages [6]. Examples include the adaptive radiation of cichlid fishes that has resulted in multiple instances of convergence in body and trophic morphology [7]; the convergent evolution of fusiform bodies with similar control surfaces (i.e., fins, flippers, flukes) in various sharks, ichthyosaurs, and cetaceans [8]; convergence of elaborate territorial displays in *Anolis* lizards on different islands in the Caribbean [9]; and the classic case of convergent morphological evolution between assorted placental and marsupial mammals that includes wolves and thylacines, cats and quolls, anteaters and numbats, mice and marsupial mice, and between placental moles (golden moles, eulipotyphlan moles) and marsupial moles [1,10] (Figure 1).

The similarities between golden moles (Chrysochloridae) and marsupial moles (Notoryctidae) are particularly striking and include the occupation of subterranean habitats; the employment of parasagittal, rapid scratch–digging for moving through the soil/sand; the presence of leathery nose pads/shields that are adapted for working the soil or pushing forward in the sand/soil; absence of the external ears; zalambdodont teeth; ears that are adapted for low-frequency hearing; and vestigial eyes that are covered by skin and hair [12,13,14]. The two extant species of *Notoryctes* (*N. typhlops*, *N. caurinus*) inhabit the sandy deserts of Australia [14]. Chrysochlorids are more diverse, and different species are found in a variety of habitats including coastal sands and sand dunes, grassy areas, alluvial sands and sandy loams within forests and savannahs, peaty soils in sheltered ravines and forests, and cultivated areas. Among chrysochlorids, the Namib Desert golden mole (*Eremitalpa granti*) is most similar to marsupial moles (*Notoryctes*) by virtue of its occupation of coastal sands and sand dunes and its ‘sand-swimming’ style of locomotion. Indeed, the similarities between chrysochlorids and *Notoryctes* are so extensive that the paleontologist Edwin Drinker Cope regarded *Notoryctes* as a “South African type of placental mammal in Australia” [15].

Chrysochloridae belongs to the order Afrosoricida along with Malagasy tenrecs and African otter shrews. There are 11 genera and 21 species of living chrysochlorids [14]. Living notoryctids comprise a monotypic family in the marsupial order Notoryctemorphia. The eyes of both chrysochlorids and notoryctids are very small and highly degenerate, but degeneracy is much more extreme in the latter with respect to most features (Table 1) [16]. In chrysochlorids, the eyes are “comparatively superficial” and located within the dermis where they are surrounded by hair roots [16]. *Notoryctes*, in turn, has eyes that are “retired far beneath the skin” and located beneath the temporalis muscle [17]. The extrinsic muscles of the eyes in *Notoryctes* are degenerative, abnormal in position, and non-striated rather than striated. In contrast, Sweet [16] reported that these muscles are completely absent in the two species of Chrysochloridae that she examined (*Chrysochloris asiatica*, *Amblysomus hottentotus*). *Notoryctes* also lacks the oculomotor (III), trochlear (IV), and abducens (VI) nerves that innervate the six extrinsic ocular muscles [17,18]. Sweet [16] reported that the oculomotor and trochlear nerves are absent in chrysochlorids, but did not comment on the presence or absence of the abducens nerve. In addition, Bhagwandin et al. [19] reported that the oculomotor and trochlear nuclei of the brain of *Chrysochloris asiatica* are present, but with far fewer nuclei than in similarly sized mammals with functional eyes. Moreover, the remaining neurons have an atypical morphology and are ovoid in shape with no clear dendrites emerging from the soma [19]. In addition, Bhadwandin et al. [19] found no evidence for the presence of an abducens nucleus in *C. asiatica*. We are unaware of equivalent studies on the oculomotor, trochlear, and abducens nuclei in *Notoryctes*, but presume that these nuclei may also be lost or reduced given the absence of the associated cranial nerves III, IV, and VI.

The optic nerve (II) and optic chiasma are also absent from the brain of *Notoryctes typhlops* [17,18]. However, remnants of this nerve, whether actual nerve fibers or just a connective tissue sheath, were found exiting the eye of one of the individuals of *N. typhlops* that Sweet [17] investigated. This sheath could not be traced to the brain. In chrysochlorid eyes (*Amblysomus hottentotus*, *Chrysochloris asiatica*), Sweet [16] found that optic nerves exiting the eye were variably present. *Eremitalpa granti* also has a degenerative optic nerve [23]. Studies of chrysochlorid brains from several species have found no trace of optic nerves or optic chiasma [20,22].

The iris and lens of the eye are degenerate but recognizable in chrysochlorids [16]; whereas in *Notoryctes,* the lens is absent and the iris is either absent or represented by only a few nuclei [17]. *Chrysochloris asiatica* eyes have a small space that Sweet [16] regarded as a potential pupil, but this structure is absent in *N. typhlops* [17]. The sclera, choroid, and cornea are indistinguishable from each other in both chrysochlorids and *N. typhlops* [16]. Importantly, the retinal layers in chrysochlorids show very little degeneration, and both types of photoreceptor cells, rods and cones, are present [16]. In contrast, both rods and cones are absent in *Notoryctes,* and the retina is an undifferentiated mass of cells [17]. Based on these comparisons, and on additional contrasts with other vertebrates that are blind or have poor eyesight including moles (*Talpa, Scapanus*), caecilians (*Siphonops*), blind snakes (*Typhlops*), and worm lizards (*Rhineura*), Sweet [17] concluded that the eyes of *Notoryctes* are significantly more degenerate than those of any other mole or terrestrial vertebrate that had been investigated and instead are most similar to the eyes of blind cavefish. (We also note that detailed anatomical studies of the eyes of many blind terrestrial vertebrates [e.g., most blind snake species] have not been reported, and it remains possible that another terrestrial vertebrate has eyes that are as degenerate as *Notoryctes*).

Darwin [24] commented on the degeneration of the eyes in subterranean mammals in his chapter that discussed the use and disuse of organs. More specifically, Darwin observed an injury to the eye of a burrowing tuco-tuco (*Ctenomys*) rodent that he kept in captivity during his time in South America, and suggested that gradual reduction in the size of the eyes with the adhesion of the eyelids and growth of fur over them might be advantageous in subterranean mammals that exhibit this condition. Along these lines, Spencer [25], p. 53 called attention to the elevated perils of Australia’s sandy deserts when he stated “It is a curious feature about *Notoryctes* that though absolutely blind still it normally spends a part of its time on the surface, and the complete loss of eyes externally is, no doubt, to be associated with the fact that it is constantly burrowing in loose and often hot sand, the grains of which would, if it had eyes, be a fruitful source of irritation”. Sweet [17] extended this line of thinking and suggested two competing hypotheses to account for the greater degeneration of the eyes in *Notoryctes* than in other moles (*Talpa*, *Scalops* [now *Scapanus*]) that had been investigated as of 1906. First, *Notoryctes* may have had more time to adapt to its burrowing life than other moles. Second, the sand in which *Notoryctes* lives may be more deleterious to the eye than is the earth in which other moles burrow. If the sandy deserts of Australia are a relatively recent development, as believed by Sweet [17], then degeneration of the eye may have been accelerated in the recent past in response to Australia’s aridification and the formation of these deserts. Indeed, recent evidence suggests that major changes to central Australia’s landscapes have taken place in the last 15 MY, and that widespread deserts and dunes may have formed as recently as the late Pleistocene and Holocene [26]. Also in support of the second hypothesis, Sweet [17] called attention to the enormous development of glandular tissue (lacrimal gland and possibly Harderian gland) in *Notoryctes* that occupies most of the eye capsules and in some individuals overflows beyond the eye capsules. Sweet [17] invoked the “law of compensation and economy of growth” and suggested that expanded glands and their secretions were necessary to (1) keep the snout and nasal cavity moist and (2) prevent the entry and accumulation of particles of sand into the nasal cavity while burrowing. Sweet [17] suggested that degeneration of the eyeball and its associated nervous structures occurred concurrently with the expansion of gland structures as a direct result of natural selection. Some chrysochlorids such as *Eremitalpa* and *Cryptochloris* also live in sandy habitats (sand dunes, coastal sands), but many chrysochlorids inhabit other substrates. The base of the crown chrysochlorid radiation has not been firmly resolved [27,28], and the early radiation of this clade may have occurred in habitats with soil rather than sand. If so, then selective pressures to modify the eyes and glands in the head may have been less severe than for the recent history of *N. typhlops*.

The discovery that DNA is the hereditary material came long after Sweet [17] outlined alternative hypotheses for the evolution of *Notoryctes typhlops*’ vestigial eyes, and phylogenomic methods can now be used to test these hypotheses. Springer et al. [29] provided molecular evidence that the gene-encoding retinol-binding protein 3 (RBP3) is a pseudogene in *Notoryctes*. RBP3 is involved in the visual cycle where it plays a role in the regeneration of vitamin-A-derived chromophores for photoreception. Sequences for genes that are involved in the rod and cone phototransduction cascades of *N. typhlops* have not been reported. The chrysochlorid *Chrysochloris asiatica*, in turn, is a rod monochromat based on sequences from its phototransduction cascade genes. Specifically, *C. asiatica* has gene sequences that encode an intact cascade of rod phototransduction activation proteins. By contrast, there are inactivating mutations in both cone opsin genes (*OPN1SW* [=*SWS1*] and *OPN1LW* [=*LWS*]) that abrogate the cone phototransduction activation cascade [30]. The chrysochlorid *Amblysomus hottentotus* also has inactivated *OPN1SW* and *OPN1LW* genes [30]. In the case of *C. asiatica,* there are also known inactivating mutations in other genes in the cone phototransduction cascade [30]. Here, we perform molecular evolutionary analyses on DNA sequences for a variety of eye-specific genes in afrotheres and marsupials to test Sweet’s [17] competing hypotheses. We chose chrysochlorids rather than the second family of placental moles, eulipotyphlan talpids, because chrysochlorids have more degenerate eyes than talpids and come closer to approximating the vestigial condition found in notoryctids. A few talpids such as *Talpa occidentalis* (Iberian mole) have eyelids that are permanently closed, but they retain a well-developed retina with rods, cones, and melanopsin-positive retinal ganglion cells that facilitate photoperiodicity [31]. Talpids also retain intact optic nerve connections between the eyes and the brain. Our sampling of candidate loci included eye-specific genes that help form the cornea and lens, as well as genes that encode proteins in the cone phototransduction pathway (activation and recovery). If the highly vestigial eyes of *N. typhlops* have degenerated over a longer timespan than the less degenerative eyes of chrysochlorids, then eye-specific genes in *N. typhlops* should be in a greater state of decay than in chrysochlorids and also show evidence for an earlier onset of relaxed selection. In contrast, if the eyes of *N. typhlops* degenerated rapidly and recently in response to increasingly sandy soil, then there may have been less time for the accumulation of inactivating mutations in notoryctid eye-specific genes than in chrysochlorid eye-specific genes. To perform these analyses, we leveraged available public genomes for 28 marsupials and 11 afrotheres and examined 13 eye-specific genes that are primarily expressed in the lens or cornea, and 10 genes that are specific to cone phototransduction. We tabulated inactivating mutations (e.g., frameshift insertions and deletions, premature stop codons), conducted selection analyses, and estimated inactivation times for the different categories of eye genes and by proxy the corresponding phenotypes of the degenerative eyes in *N. typhlops* and chrysochlorids. Given the absence of an optic nerve connection to the brain in chrysochlorids and *N. typhlops*, as well as the loss of rods and cones in *N. typhlops*, we also examined nine genes that are hypothesized to be specific to rod phototransduction to determine if any of these genes exhibit inactivating mutations in *N. typhlops* and/or chrysochlorids. In addition to assembled genomes, we generated new genomic sequence data for the chrysochlorid *A. hotttentotus* (Hottentot golden mole) and a second individual of *N. typhlops* to complement the assembled *N. typhlops* genome on DNA Zoo (https://www.dnazoo.org). Finally, we integrate our molecular results with information from the fossil record for both chrysochlorids and notoryctids.

## 2. Materials and Methods

### 2.1. Gene Sampling

We targeted 38 eye genes, of which the majority are eye-specific, and at least six appear to be pleiotropic. The pleiotropic genes serve as baseline controls for which we do not expect to find inactivating mutations in *Notoryctes* or either of the chrysochlorids. The 38 eye genes are distributed across three general categories: cone phototransduction (12 genes), rod phototransduction (12 genes), and lens/cornea development (14 genes). For both the cone phototransduction pathway and the rod phototransduction pathway, we included genes that are associated with the activation and recovery phases of phototransduction. The activation genes encode proteins that facilitate the four basic steps of the phototransduction cascade: (1) activation of G protein-coupled receptors (opsins) following the absorption of light by the chromophore 11-cis retinal, (2) activation of the G protein transducin, (3) activation of phosphodiesterase (PDE proteins), and (4) hyperpolarization of the photoreceptor cell following closure of the sodium channels by cGMP-gated channels [32]. The recovery genes, in turn, encode proteins that are associated with shut-off (G protein-coupled receptor kinases, arrestins) [33] and Ca^2+^ feedback regulation (sodium/calcium–potassium exchangers) [34]. With one exception, these genes correspond to the cone-only and rod-only categories of Invergo et al. [35]. The exception is *GRK1*, which Lamb [36] included in the rod-only category instead of the shared category of Invergo et al. [35]. *GRK1* is known to be expressed in the cones of both primate and rodent species in the superorder Euarchontoglires, but not in pig and dog species that belong to the superorder Laurasiatheria [37]. We are unaware of *GRK1* or *GRK7* expression studies in marsupials or afrotheres. The complete list of cone and rod genes is as follows: cone genes (*ARR3*, *CNGA3*, *CNGB3*, *GNAT2*, *GNB3*, *GNGT2*, *GRK7*, *OPN1LW* (=*LWS*), *OPN1SW* (=*SWS1*), *PDE6C*, *PDE6H*, *SLC24A2*); rod genes (*CNGA1*, *CNGB1*, *GNAT1*, *GNB1*, *GNGT1*, *GRK1*, *PDE6A*, *PDE6B*, *PDE6G*, *RHO* (=*OPN2*), *SAG*, *SLC24A1*). Note that two genes in the cone phototransduction pathway (*GNB3*, *SLC24A2*) and at least three genes in the rod phototransduction pathway (*CNGB1*, *GNB1*, *RHO*) appear to be pleiotropic and are not expected to exhibit inactivating mutations. In the case of *GNB3*, ablation of this gene results in brachycardia in mice [38]. Overexpression of *GNB3* in mice leads to reduced expression of *UCP1* and obesity [39]. In humans, polymorphic SNPs in *GNB3* are associated with adverse reactions to antidepressants [40] and hypertension susceptibility [41]. The *SLC24A2* gene, in turn, may influence the function of islet *β* cells in the pancreas [42]. In the rod phototransduction activation pathway, *GNB1* encodes a transducin subunit in the rod phototransduction pathway that performs a similar function to the GNB3 protein in the cone pathway. Known *GNB1* mutations in humans are associated with severe neurodevelopmental delay, abnormal muscle tone, and genitourinary anomalies in males [43,44,45,46]. In addition, an alternative transcript of *CNGB1* encodes a protein (CNGB1b) that is a component of CNG channels in the olfactory transduction cascade [47]. Finally, *RHO* is expressed in the skin [48,49,50] and also plays a role in sperm thermotaxis [51,52].

Our gene sampling also included 14 genes that are primarily related to the lens and/or cornea, including eight crystallin genes, of which two encode alpha crystallins (*CRYAA*, *CRYAB*), five encode beta crystallins (*CRYBA1*, *CRYBA4*, *CRYBB1*, *CRYBB2*, *CRYBB3*), and one encodes a gamma crystallin (*CRYGS*) [53]. With one exception (*CRYAB*), these crystallins are primarily or exclusively expressed in the lens, although in some cases they have additional functions in other parts of the eye. *CRYAA* [54] is also expressed in the retina; mutations in human *CRYBA4* are associated with microphthalmia [55] and microcornea [56]; and human *CRYBB1* mutants are associated with congenital cataracts as well as microcornea [57]. In contrast with these eye-specific crystallins, *CRYAB* is expressed in many other tissues including muscle [53,58]. As for other pleiotropic genes (*CNGB1*, *GNB1*, *GNB3*, *RHO*, *SLC24A1*), the inclusion of *CRYAB* provides a baseline control for which we did not expect to find inactivating mutations in *Notoryctes* or either of the chrysochlorids. Other lens- and cornea-specific genes that we targeted included *BFSP1*, *BFSP2*, *GJA8*, *LIM2*, *MIP*, and *KRT12*. *BFSP1* and *BFSP2* encode beaded-filament structural proteins that are exclusively expressed in the fiber cells of the eye lens where they contribute to the cytoskeletal network of these cells [59]. Two other genes, *GJA8* and *LIM2*, are associated with intercellular communication between the lens epithelium and lens fiber cells [60]. *MIP* encodes the major intrinsic protein of the lens fiber cells and comprises more than 60% of the membrane protein [61]. *MIP* belongs to the aquaporin family and is also known as *AQP0*. MIP acts as a water channel and plays a structural role where it helps to maintain the transparency and optical accommodation of the lens [62]. Finally, *KRT12* is a keratin gene that is expressed in the corneal epithelium [63,64]. Accession numbers for all of the eye-gene sequences that were employed in our study, including accession numbers from NCBI’s Sequence Read Archive (SRA) that were used to fill in or correct genomic sequences, are provided in the protein-coding sequence alignments in the supplementary information. An example of a genome sequence that was corrected is a 1 bp frameshift deletion in exon 1 of the *OPN1LW* gene in the genome sequence of *Phascolarctos cinereus* (NW_018344262). This putative inactivating mutation is actually an assembly error based on 10X coverage in the SRA (ERR1881585-ERR1881587) that instead validates the coding integrity of exon 1 in *P. cinereus*.

### 2.2. Taxon Sampling

Taxon sampling for marsupials included 28 taxa with assembled genomes. These taxa index five of seven marsupial orders as follows: Didelphimorphia (*Didelphis virginiana* (Virginia opossum), *Gracilinanus agilis* (agile gracile opossum), *Monodelphis domestica* (gray short-tailed opossum)); Microbiotheria (*Dromiciops gliroides* (monito del monte)); Diprotodontia (*Vombatus ursinus* (common wombat), *Phascolarctos cinereus* (koala), *Trichosurus vulpecula* (brushtail possum), *Strigocuscus gymnotis* (ground cuscus), *Petaurus breviceps* (sugar glider), *Pseudochirops corinnae* (plush-coated ringtail possum), *Pseudochirops cupreus* (coppery ringtail possum), *Pseudocheirus occidentalis* (western ringtail possum), *Potorous gilbertii* (Gilbert’s potoroo), *Setonix brachyurus* (quokka), *Lagorchestes hirsutus* (rufous hare-wallaby), *Macropus eugenii* (tammar wallaby), *Macropus fuliginosus* (western gray kangaroo), *Macropus giganteus* (eastern gray kangaroo), *Macropus rufus* (red kangaroo)); Dasyuromorpha (*Thylacinus cynocephalus* [thylacine], *Myrmecobius fasciatus* (numbat), *Sarcophilus harrisii* (Tasmanian devil), *Dasyurus hallucatus* (northern quoll), *Dasyurus viverrinus* (eastern quoll), *Phascogale tapoatafa* (brush-tailed phascogale), *Antechinus flavipes* (yellow-footed antechinus), *Antechinus stuartii* (brown antechinus)); Notoryctemorphia (*Notoryctes typhlops* (southern marsupial mole)). Whole-genome Illumina data were also generated for a second individual of *N. typhlops* (see below). Taxon sampling for afrotheres included 11 species with assembled genomes. These species index all six orders in Afrotheria as follows: Afrosoricida (*Chrysochloris asiatica* (Cape golden mole), *Echinops telfairi* (lesser hedgehog tenrec), *Microgale talazaci* (Talazac’s shrew tenrec)); Hyracoidea (*Heterohyrax brucei* (bush hyrax), *Procavia capensis* (rock hyrax)); Macroscelidea (*Elephantulus edwardii* (Cape elephant shrew)); Proboscidea (*Elephas maximus* (Asian elephant), *Loxodonta africana* (African savannah elephant)); Sirenia (*Dugong dugon* (dugong); *Trichechus manatus* (West Indian manatee)); Tubulidentata (*Orycteropus afer* (aardvark)). In addition, whole-genome Illumina data were generated for a second chrysochlorid, *Amblysomus hottentotus* (Hottentot golden mole). 

### 2.3. BLAST Searches and Data Collection

DNA sequences were obtained from (1) assembled genomes at NCBI (https://www.ncbi.nlm.nih.gov/) and DNA Zoo (https://www.dnazoo.org), (2) raw sequence reads at NCBI’s Sequence Read Archive (SRA) (https://www.ncbi.nlm.nih.gov/sra), and (3) newly generated Illumina whole-genome sequence data for *Amblysomus hottentotus* and *Notoryctes typhlops*. NCBI’s Nucleotide database was searched using keywords for all 38 gene and taxon names for reference species (usually *Bos taurus*, *Homo sapiens*, *Loxodonta africana*, *Trichechus manatus*, *Phascolarctos cinereus*, *Trichosurus vulpecula*). Sequences for each reference species and gene were imported into Geneious Prime (current version 2023.0.2 Build 9 January 2023 11:52, https://geneious.com) [65], aligned with MAFFT v7.490 [66,67,68], and then cross-checked against each other for consistent exon annotations. In cases where there were inconsistent annotations, we examined annotated sequences for additional species. For 34 of 38 genes, we recognized the same exons in *B. taurus*, *H. sapiens*, both afrotheres, and both marsupials. The exceptions are *ARR3*, *CNGA3*, *CNGB1*, and *SLC24A1*. *ARR3* is a pseudogene in afrotheres [30], and we ignored any remnants of this gene in this taxon. *CNGA3* has an extra coding exon (exon 5 of eight coding exons) in *Bos taurus* that is also annotated in *Trichosurus vulpecula*. This exon is present in all marsupial genomes that we investigated and was incorporated into our marsupial *CNGA3* alignment. For *CNGB1*, placental mammals have 32 exons, whereas there are only 18 exons in marsupials. Marsupials lack the first 15 coding exons of placentals, and the first marsupial exon is orthologous, at least in part, to a region of intron 15 in placentals. The putative 1st exon of *CNGB1* is 223 bp in all 28 marsupials without any length variation. Finally, *SLC24A1* has eight exons in marsupials and nine exons in placentals. Exons 1–3 and 6–9 in placentals have clear orthologous relationships to exons 1–3 and 5–8 in marsupials, but exons 4 and 5 in placentals do not have orthologous protein-coding exons in marsupials. Exon 4 in marsupials, in turn, does not have an orthologous protein-coding exon in afrotheres. Nevertheless, exon 4 in marsupials is 104 bp in all 28 taxa without any length variation. Sequences for additional species were harvested with NCBI’s Nucleotide Basic Local Alignment Search Tool (BLAST), which was used online for sequences on NCBI’s RefSeq or whole-genome shotgun (WGS) databases), and in Geneious Prime for genomes that were downloaded from DNA Zoo and formatted into searchable databases in Geneious Prime. We also used BLAST to search unassembled genome sequences in NCBI’s SRA database. Each BLAST search employed a query sequence from a closely related species. Megablast was used for highly similar sequences (e.g., taxa in same genus of family); blastn was used for less similar sequences (e.g., taxa in different families or orders). Top-scoring results from megablast and blastn searches were imported into Geneious Prime. Sequences that were obtained through NCBI’s SRA database were assembled using Geneious Prime’s ‘Map to Reference’ approach, where the reference sequence was either the same taxon or a closely related species to the SRA taxon. We used the same taxon in cases where inactivating mutations in genome sequences needed to be checked and a closely related taxon when we were attempting to fill in a missing exon or portion thereof. We also used the map-to-reference approach in Geneious Prime to assemble genes of interest for two samples (*Notoryctes typhlops*, *Amblysomus hottentotus*) for which we generated paired-end Illumina sequence reads (see below). We allowed for a maximum mismatch of 5% per read (same taxon) or 15% per read (different taxa) and required a minimum of two reads for base calling with a consensus threshold of 50%.

### 2.4. New Genome Data

Unassembled genome sequences for *Notoryctes typhlops* and *Amblysomus hottentotus* were obtained from DNA libraries that were constructed with Illumina’s NeoPrep procedure after sonicating the samples to a mean length of 550 bp at the University of California, Riverside (UCR), Genomics Core Facility. These libraries were then sequenced at ~35–40X coverage at the New York Genome Center using paired-end sequencing (150 bp per read) on a HiSeq 2500 platform. The sample for *N. typhlops* was originally provided to Dr. Michael Westerman (LaTrobe University) in 1992 by CSIRO. Dr. Carey Krajewski extracted DNA from this sample in the same year. The sample for *A. hottentotus* (T-1903) was a gift from Dr. John Kirsch (University of Wisconsin, Madison) to M.S.S. in 1996. Dr. Kirsch, in turn, received the sample from Dr. Charles Sibley (Yale University).

### 2.5. Alignments and Tabulation of Inactivating Mutations

Complete protein-coding sequences (CDS) and introns were aligned in Geneious Prime using MAFFT v7.490 [66,67,68] and MUSCLE 5.1 [69]. Sequence alignments were manually inspected by eye and adjusted if necessary. Sequences were annotated in Geneious Prime for exons and various types of inactivating mutations (frameshift insertions and deletions, start and stop codon mutations, donor and acceptor splice-site mutations, intron–exon boundary deletions). We also created a version of each alignment that only contained exons to screen for premature stop codons (frameshift insertions were deleted and frameshift deletions were filled with Ns prior to performing this screen). Furthermore, we examined key functional sites in the protein sequences of the opsins (*OPN1SW*, *OPN1LW*, *RHO*), including spectral tuning sites, the seven transmembrane domains, the chromophore binding site, and the residue that provides the Schiff base counter ion [70,71]. The transmembrane regions are based on GenBank annotations referencing mRNAs (*Bos taurus*). Mutations were mapped onto species trees for Afrotheria and Marsupialia using delayed transformation (DELTRAN) parsimony optimization.

Given the possibility of genome assembly errors, it is important to validate inactivating mutations. Inactivating mutations that are shared by both chrysochlorid species or both individuals of *Notoryctes typhlops* were validated by their dual occurrence. Autapomorphic inactivating mutations in *Amblysomus hottentotus* and the NYGC individual of *N. typhlops* were validated by their occurrence in multiple Illumina reads. For autapomorphic inactivating mutations in *Chrysochloris asiatica* and the DNA Zoo individual of *N. typhlops*, we validated these mutations by BLASTing (megablast) genomic segments containing these mutations to Illumina reads on NCBI’s Sequence Read Archive (Table S1).

### 2.6. Phylogenetic Analyses

Maximum-likelihood gene trees were reconstructed from complete protein-coding sequences (Supplementary Information) using RAxML 8.2.11 [72] with the GTRGAMMA option in Geneious Prime. Analyses were performed with rapid bootstrapping (500 pseudoreplications) and a search for the best-scoring maximum-likelihood tree in the same search [73]. We also performed analyses on concatenations of 38 eye genes (37 for Afrotheria) with RAxML. The concatenated data sets included 50,206 nucleotide positions for afrotheres and 50,217 nucleotide positions for marsupials. Analyses were performed with a different partition for each gene. The concatenation species trees were rooted between Didelphimorphia and other taxa (marsupials) or between Paenungulata and Afroinsectiphilia (afrotheres) [11,74].

### 2.7. Selection Analyses

Selection (dN/dS) analyses were performed with the codeml program in PAML 4.4 [75]. We performed branch analyses with separate branch categories for background branches, transitional branches, and fully pseudogenic branches [3]. Background branches are functional branches that lack inactivating mutations; transitional branches record the first inactivating mutation in a lineage; and fully pseudogenic branches postdate the first occurrence of an inactivating mutation on an earlier branch. Transitional branches were stem Chrysochloridae (all afrothere analyses) and stem *Notoryctes typhlops* (all marsupial analyses). The pseudogenic categories were crown Chrysochloridae and crown *Notoryctes typhlops* (two individuals). For both data sets, we performed analyses with estimated and fixed (dN/dS = 1.0) values for the fully pseudogenic branch category. Selection analyses were performed with separate concatenations of eye-specific genes as follows: eight cone phototransduction activation genes, one (Afrotheria) or two (Marsupialia) cone phototransduction recovery genes, six rod phototransduction activation genes, three rod phototransduction recovery genes, and 12 (Afrotheria) or 13 (Marsupialia) lens/cornea genes (Table 2 and Table 3). Note that six genes that are presumed to be pleiotropic (*CNGB1*, *CRYAB*, *GNB1*, *GNB3*, *RHO*, *SLC24A2*,) were omitted from the concatenated sets of eye-specific genes. For the afrothere analysis with cone phototransduction recovery genes, there was only one gene because *ARR3* is a pseudogene in Afrotheria [30]. For analyses with the marsupial cone phototransduction activation data set, we omitted *Strigocuscus gymnotis OPN1SW* because this gene sequence is pseudogenized, and we infer that *S. gymnotis* is an L-cone monochromat (see Results). For analyses with the marsupial rod phototransduction recovery data set, we added an extra transitional branch category for *Myrmecobius fasciatus* because two of the recovery genes (*GRK1*, *SLC24A1*) are pseudogenized in this taxon. For analyses with the afrothere cone phototransduction recovery data set, we added an extra transitional branch category for *Echinops telfairi* because one of the two recovery genes is pseudogenized in this taxon. For analyses with the afrothere lens/cornea data set, we omitted *CRYBA4* because there were no BLAST results for this gene in either of the chrysochlorids. We also excluded the *CRYBB1* and *CRYBB3* sequences for tenrecs because these genes are either pseudogenized (*E. telfairi CRYBB1* and *CRYBB3*; *Microgale talazaci CRYBB1*) or returned no BLAST results (*M. talazaci CRYBB3*). We presume that *CRYBB1* and *CRYBB3* do not participate in the formation of the lens in these two tenrecs. We performed analyses with two codon frequency models (CF1, CF2) in codeml. We employed both models because codon positions are present in taxa with functional copies of genes but absent in taxa with pseudogenes. It is, therefore, important to verify those analyses without base compositional differences at 1st, 2nd, and 3rd codon positions (CF1) yield similar results to those obtained with a more sophisticated codon frequency model (CF2) that allows for base compositional differences at 1st, 2nd, and 3rd codon positions [76]. Analyses with codeml were performed after deleting frameshift insertions and replacing premature stop codons with NNN. We used species trees from Meredith et al. [11] for afrotheres and Duchêne et al. [77] for marsupials. For marsupials that were not included in Duchêne et al.’s [77] data set, we added these taxa to the marsupial tree based on our maximum-likelihood analysis with the concatenated data set of 38 genes (see Results).

### 2.8. Estimation of Gene Inactivation Times

We used equations from Meredith et al. [3] and divergence times from Meredith et al. [11] for afrotheres and Duchêne et al. [77] for marsupials to estimate the onset of relaxed selection (=neutral evolution) for groups of eye-specific genes, and by proxy the associated phenotypes, in Chrysochloridae and *Notoryctes typhlops*. Meredith et al. [11] estimated a divergence time of 68.21 million years ago (MYA) for the split between Chrysochloridae and Tenrecidae, and a divergence time of 12.04 MYA for the split between *Chrysochloris asiatica* and *Amblysomus hottentotus*. Duchêne et al. [77] estimated a divergence time of 63.39 MYA for the split between Notoryctemorphia and Dasyuromorpha. To obtain dN/dS estimates for the fully pseudogenic branch category of *N. typhlops,* it was necessary to include both individuals in the analyses. We estimated a split between the two individuals of *N. typhlops* by solving for the unknown in the following proportionality that assumes a molecular clock: unknown split between *N. typhlops* individuals/known split between Notoryctemorphia and Dasyuromorphia at 63.39 MYA = mean branch length for terminal branches leading to *N. typhlops* individuals on the ML concatenation tree (0.000727 substitutions/site)/mean branch length for stem + crown *N. typhlops* branches on the ML concatenation tree (0.072027 substitutions/site). This yielded an estimate of 0.64 MYA for the split between the two *N. typhlops* individuals. We estimated inactivation times for four groups of genes as follows: cone phototransduction activation (eight genes), cone phototransduction recovery (two genes in *N. typhlops*, one gene in Chrysochloridae (no *ARR3*)), rod phototransduction recovery (three genes), and lens + cornea (thirteen genes in *N. typhlops*, twelve genes in Chrysochloridae (no remnants of *CRYBA4* were recovered in Chrysochloridae)). By combining individual genes into groups of genes that are associated with a related function, we were able to achieve more statistical power than is possible with individual genes [78,79]. This approach assumes that groups of eye-specific genes have evolved under neutral selection that is coincident with ablation of function, even in cases where the accumulation of an inactivating mutation (e.g., frameshift indel, premature stop codon) in one or more genes has lagged behind the jobless role of that gene(s) [78]. In addition, we estimated a possible inactivation time for rod phototransduction activation genes in *N. typhlops*. A caveat is that we detected an inactivating mutation(s) in only one of six genes that are presumed to be specific for rod phototransduction. Further, this mutation is only present in one of two individuals of *N. typhlops*. We estimated inactivation times using eight different combinations of codon frequency model (CF1 or CF2), estimated versus fixed (1.0) values for dN/dS (=ω) on fully pseudogenic branches, and two different equations that allow for one versus two synonymous rates of substitution [3,78,79].

## 3. Results

### 3.1. Alignments, Gene Trees and Species Trees

Protein-coding-sequence (CDS) alignments for individual genes, maximum-likelihood (RAxML) gene trees, and partitioned maximum-likelihood (RAxML) species trees based on concatenations of phototransduction + lens/cornea genes are provided in the supplementary information. For Afrotheria, all 37 gene trees with the required taxon sampling are consistent with the monophyly of Hyracoidea, Proboscidea, Sirenia, Chrysochloridae, and Tenrecidae. The concatenation tree for Afrotheria is generally consistent with higher level relationships based on multigene and phylogenomic data sets [11,74], including a basal split between Paenungulata (Hyracoidea + Proboscidea + Sirenia) and Afroinsectiphilia (Afrosoricida + Macroscelidea + Tubulidentata) and the monophyly of Afroinsectivora (Afrosoricida + Macroscelidea). However, Afrosoricida was recovered as paraphyletic with Macroscelidea nested inside of Afrosoricida as the sister taxon to Tenrecidae. For Marsupialia, the 38 gene trees are largely consistent with the monophyly of well-established marsupial clades ranging from tribes to superorders: Australidelphia (38/38) Dasyurini (37/38), Dasyuridae (38/38), Dasyuromorphia (38/38), Didelphimorphia (38/38), Diprotodontia (38/38), Macropodidae (36/38), Macropodoidea (37/38), Petauroidea (36/38), Phalangeridae (36/38), Phalangeriformes (28/38), Phascogalini (33/38), Pseudocheiridae (32/38), and Vombatiformes (35/38). The concatenation species tree for marsupials supports each of these clades, as well as the monophyly of Australasian marsupials. In all cases where there is overlapping taxon sampling with Duchêne et al.’s [77] phylogenomic data set, which is comprised of 1550 loci and 867,000 aligned nucleotide sites, there is 100% topological agreement.

### 3.2. Gene Inactivation in the Marsupial Mole

**Figure 2:**
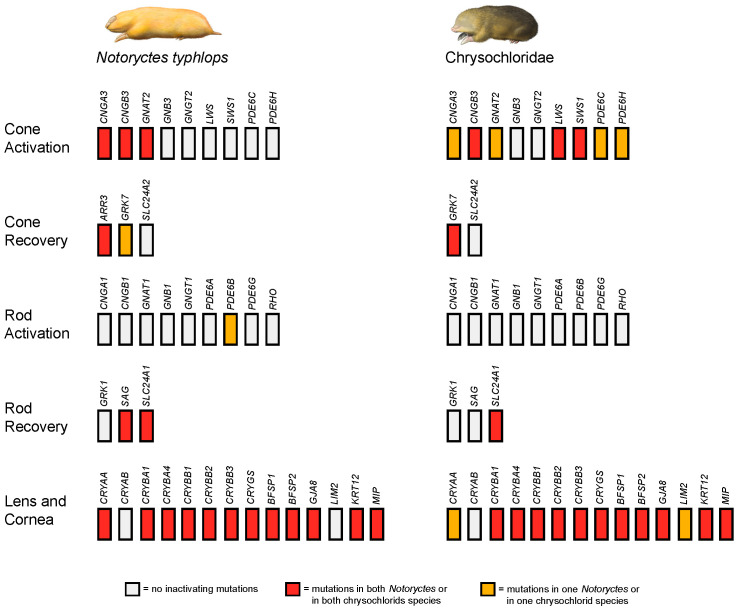
Summary of genes with and without inactivating mutations in *Notoryctes typhlops* (**left**) and two species of Chrysochloridae (*Amblysomus hottentotus*, *Chrysochloris asiatica*) (**right**). Vertical rectangles with light-gray shading indicate genes for which there are no inactivating mutations in both individuals of *N. typhlops* or both species of Chrysochloridae. Vertical rectangles with yellow shading indicate genes for which there is an inactivating mutation(s) in one of the two individuals of *N. typhlops* or one species of Chrysochloridae. Vertical rectangles with red shading indicate genes for which there is an inactivating mutation(s) in both individuals of *N. typhlops* or both species of Chrysochloridae.

**Figure 3:**
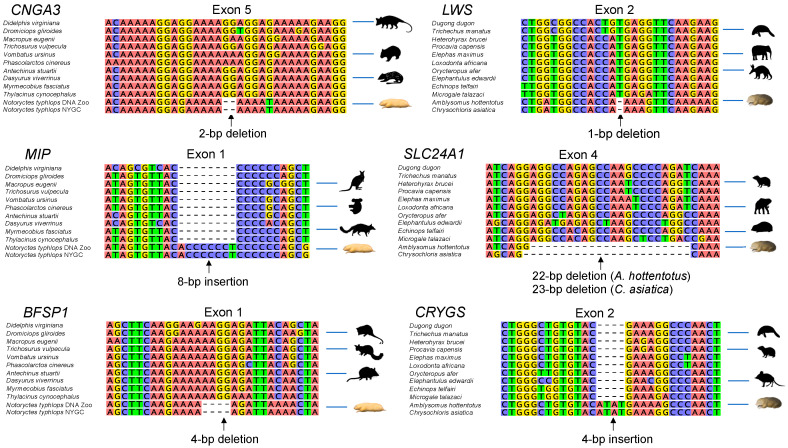
Examples of inactivating mutations in *Notoryctes typhlops* (**left**) and chrysochlorids (**right**). Gene names are shown above taxon names, and exon numbers are shown above alignments. Only 11 of 28 marsupial species are shown. Silhouettes are for the same species or a closely related species.

Inactivating mutations in the cone and rod phototransduction genes of *Notoryctes typhlops* are provided in Table 2. Inactivating mutations in the lens/cornea genes of *N. typhlops* are listed in Table 3. Figure 2 provides a visual summary of eye genes that are intact or have inactivating mutations in *N. typhlops*. Figure 3 provides examples of inactivating mutations in *N. typhlops*. Both cone opsin (*OPN1LW*, *OPN1SW*) genes have intact coding sequences, splice sites (canonical), spectral tuning sites, transmembrane domain regions, and chromophore binding and Schiff base counterion sites. However, three of eight genes that are specific for cone phototransduction activation (*CNGA3*, *CNGB3*, *GNAT2*) exhibit inactivating mutations that are shared by both individuals of *N. typhlops* (Table 2). *GNB3*, which is pleiotropic and has an additional role outside of phototransduction [40,41], is intact in both *N. typhlops* individuals. Among the three cone phototransduction recovery genes, *ARR3* exhibits multiple inactivating mutations in both individuals; *GRK7* displays a premature stop codon in one of the two *N. typhlops* individuals; and the pleiotropic *SLC24A2* gene is intact in both individuals.

**Table 2:** Inactivating mutations * in cone and rod phototransduction genes in *Notoryctes typhlops* and two species of Chrysochloridae (*Chrysochloris asiatica, Amblysomus hottentotus*). Reported results for *N. typhlops* are for both individuals (DNA Zoo, NYGC) unless otherwise noted. Numerical positions refer to coding sequence alignments.

Gene (# of Marsupial Exons/# of Afrothere Exons)	Inactivating Mutations			
	*Notoryctes typhlops*	Chrysochloridae	*C. asiatica*	*A. hottentotus*
Cone Phototransduction Activation				
*CNGA3* (8/7)	471–472D (E5)	None	None	2074–2076S (E7 ^A^)
*CNGB3* (18/18)	1000D (E9, NYGC only) 1478–1575D (E13), 1654–1658I (E14), 1823–1827D (E16)	1811–1813S (E16), 2069–2071S (E17)	In1Do (AT), 655–657S (E6), 751–753S (E6), In8Ac (GG), 1000–1002S (E9), 1147–1149S (E10), 1192–1194S (E11), In12Do (AT), 1874–1876S (E16), 1955D (E17), 2021–2023S (E17), 2086D (E17), 2107–2108D (E18), 2460I (E18)	451–453S (E4), 640–642S (E6), 1404–1407D (E12), 1659D (E14), 1759I (E15), 2085–2088D (E17), 2213–2232D (E18), 2242–2427I (E18)
*GNAT2* (8/8)	1–118D (E1), 135–136D (E2)	None	1–118D (E1 ^B^), 355–356D (E4), 827I (E7), 887–888D (E8), 974–976S (E8)	None
*GNB3*^#^ (9/9)	None	None	None	None
*GNGT2* (2/2)	None	None	None	None
*OPN1LW* (=*LWS*) (6/6)	None	180–182S (E2), 241D (E2), 1043D (E6)	1–3SCM (E1), 390–392SBCM (E2), 556–560D (E3), In4E5BD (97 bp of In4 and 1 bp (750) of E5), E5In5BD (6 bp (984–989) of E5 and 33 bp of In5), 1027I (E6)	In1E2BD (last 12 bp of In1 and first 13 bp (116–128) of E2), 142D (E2), 163–164I (E2), 286–326D (E2), 479D (E3), In4Do (TT), In4E5BD (121 bp of In4 and 22 bp (750–771) of E5), 1026D (E6), 1099–1101TCM (E6)
*OPN1SW* (=*SWS1*) (5/5)	None	263–271STSD (E1)	773–774D (E4)	219I (E1), 388–389D (E2), 710–712S (E4), 878–880CBSM (E4), 932–934S (E5)
*PDE6C* (22/22)	None	None	3D (E1), 175D (E1), 634–636S (E2), In10Do (GG), 1109–1112I (E8), 1754–1863D (E14), 1943–1945S (E15), 2624–2626TCM (E22)	None
*PDE6H* (3/3)	None	None	In2Ac (AA), 240–252D (E3, also 27 bp in 3′ UTR)	None
2. Cone Phototransduction Recovery				
*ARR3* (16/0)	1075D (E14), In14Do (CT), E15In15BD (2 bp (1084,1085) of E15, 7 bp of In15), In15Ac (AC), 1164D (E16)	Pseudogene in Afrotheria	Pseudogene in Afrotheria	Pseudogene in Afrotheria
*GRK7* (4/4)	1306–1308S (E3, DNA Zoo only)	1567–1569S (E4), 1631–1662D (E4)	1–1060NBR/NMR (E1–2), 1234–1237I (E3), 1365D (E4)	1–3SCM (E1), 13–16I (E1), 497–499S (E1), 936D (E2), 962D (E2), 1236–1237I (E3)
*SLC24A2*^#^ (10/10)	None	None	None	None
3. Rod Phototransduction Activation				
*CNGA1* (8/8)	None	None	None	None
*CNGB1* (18/32)	None	None	None	None
*GNAT1* (8/8)	None	None	None	None
*GNB1*^#^ (9/9)	None	None	None	None
*GNGT1* (2/2)	None	None	None	None
*PDE6A* (22/22)	None	None	None	None
*PDE6B* (22/22)	In6Do (AT, NYGC only)	None	None	None
*PDE6G* (3/3)	None	None	None	None
*RHO* (5/5)	None	None	None	None
4. Rod Phototransduction Recovery				
*GRK1* (7/7)	None	None	None	None
*SAG* (15/15)	8–23D (E1), 173–366D (E4), 412–414S (E5, NYGC only), 805I (E10), 1106–1108S (E15, NYGC only)	None	None	None
*SLC24A1* (8/9)	408D (E1, NYGC only), 414I (E1), 515–517S (E1), 1634I (E1), 1670D (E1), 2387–2684D (E5), 4293–4295S (E8)	None	2110–2132D (E4)	2111–2132D (E4)

Abbreviations: Ac, acceptor splice site; BD, boundary deletion; CBSM, chromophore binding-site mutation; D, deletion; Do, donor splice site; E, exon; I, insertion; In, intron; NBR, No BLAST results; NMR, no mapped reads; S, premature stop codon; SBCM, Schiff base counterion mutation; SCM, start codon mutation; STSD, spectral tuning site deletion; TCM, termination codon mutation; and UTR, untranslated region. * Locations of inactivating mutations in exons are based on alignments for complete protein-coding sequences including frameshift insertions when present. # Genes that are pleiotropic rather than eye specific. ^A^ Premature stop is only two codons upstream from the canonical stop and may not impact the function of the protein. ^B^ Exon 1 is deleted based on an alignment of *Elephas maximus* and *Chrysochloris asiatica* that includes *AMPD2* and *GNAT2* (*AMPD2* is upstream of exon 1 of *GNAT2* in both species). Also, the map to reference results for Illumina reads for *C. asiatica* (SRR387338, SRR387339, SRR387340, SRR387341, SRR387343, SRR387345, SRR387346, SRR387349, SRR387350, SRR387352, SRR387353) with an exon 1 probe sequence from *Amblysomus hottentotus GNAT2* retrieved exon 1 of *GNAT1* but nothing for *GNAT2*.

**Table 3:** Inactivating mutations* in eye lens/cornea genes for *Notoryctes typhlops* and two species of Chrysochloridae (*Chrysochloris asiatica, Amblysomus hottentotus*). Reported results for *N. typhlops* are for both individuals (DNA Zoo, NYGC) unless otherwise noted. Numerical positions refer to coding sequence (CDS) alignments.

Gene (# of Marsupial Exons/# of Afrothere Exons)	Inactivating Mutations			
	*Notoryctes typhlops*	Chrysochloridae	*C. asiatica*	*A. hottentotus*
Alpha crystallins				
*CRYAA* (3/3)	376–378S (E3)	None	In1Do (CT)	None
*CRYAB* (3/3)	None	None	None	None
2. Beta crystallins				
*CRYBA1* (6/6)	91–93S (E2), 324–330D (E4), 405–412D (E5), 433–435S (E5), 466D (E5), 593I (E6, NYGC only), 625I (E6), 646–648TCM (E6)	174–216D (E3), 502–649NBR/NRM (E6)	84I (E2), 168–169D (E3), 254–256S (E4), 420D (E5), 483–495S (E5), In5Do (TT)	1–3SCM (E1), 359–501NRM (E5), 570–573D (E6), 586–589D (E6), 647–649TCM (E6)
*CRYBA4* (5/5)	307–309S (E4, NYGC only), 427–429S (E4)	NBR/NMR	None	None
*CRYBB1* (5/5)	1–3SCM (E1), 203D (E2), 301–303S (E3), 385–387S (E3), 523–525S (E4), In4Do (AT)	232–234S (E2), 542–546D (E4), 569–570D (E4), 575–762NBR/NMR (E5)	14D (E1), 194–212D (E2), 518D (E4), In4Do (AT)	328D (E3), 441–451D (E4), In4Do (AG)
*CRYBB2* (5/5)	In2Ac (GG, NYGC only), 241–243S (E3), In4Do (AT)	None	25–51D (E1), 252–267D (E3), 397D (E4), 467D (E5), 619–621TCM (E5)	In1Do (GG), 109–128D (E2)
*CRYBB3* (5/5)	439–441S (E4, DNA Zoo only), 635–636TCM (E5)	In1E2BD (14 bp of I1 and 9 bp [77,78,79,80,81,82,83,84,85] of E2), 177D (E2), 265–267S (E3), 437–439S (E4), 638–640TCM	115–117S (E2), 129–132D (E2), 418–420S (E4), 443–453D (E4)	293–294D (E3)
3. Gamma crystallins				
*CRYGS* (3/3)	19–21S (E1, NYGC only), 121–123S (E2), 267–297D (E3), 511–513S (E3)	151–154I (E2), 413–415S (E3), 492D (E3), 519–526I (E3)	200–202S (E2)	194–196S (E2), 244D (E2)
4. Other lens/cornea genes				
*BFSP1* (8/8)	355–357S (E1), In1Ac (A-), 424–519NBR/NMR (E3), In5Ac (TG), 1105–1108D (E8), 1255–1257S (E8), 1582–1584S (E8), 1642–1644S (E8), 1725D (E8)	16I (E1), 356D (E1), In1Ac (TG), 538–540S (E3)	366–367D (E1), E2INV ^A^, 409–411S (E2), 530D (E3)	38–102D (E1), 279–289I (E1), 343–345S (E1), 366–375D (E1)
*BFSP2* (7/7)	145D (E1), 466–548NBR/NMR (E2), 571–573S (E3), 868–999NBR/NMR (E5), In6Ac (GA)	None	999–1002I (E5), In5Do (GG), 1120–1121I (E6), 1164I (E6), 1193D (E6), In6Do (AT)	1180–1181D (E6)
*GJA8* (1/1)	302D (E1), 561–562I (E1), 980–983D (E1)	None	22–198AfSI (E1), 309–311S (E1), 889I (E1), 991–997D (E1), 1311–1314D (E1), 1524–1525D (E1), 1555D (E1)	NRM ^B^
*LIM2* (4/4)	None	None	1–3SCM (E1), 156–160I (E1), 162–164S (E1)	None
*MIP* (4/4)	62D (E1), 324–331I (E1), 567–569S (E3)	In3Ac (GG), 663–665S (E4), 708–711D (E4)	165D (E1), 426–428S (E2), 788D (E4)	328–335I (E1), In2Do (AT), 556–574D (E3), 764–788D (E4)
*KRT12* (8/8)	774D (E2), In2Ac (AC), 1108–1110S (E4), 1450I (E6)	250–272D (E1), 454–456S (E1), 523–524D (E1),	In2Ac (GG), 637–639S (E3), 692–698D (E3), 863–872I (E4), 1003I (E5), 1238I (E6), 1322I (E6), In6Ac (CG), 1415–1417S (E8)	17–20D (E1), 535–537S (E1), In1Ac (Ac), 638–630D (E3), 945I (E4), In7Ac (GG),

Abbreviations: Ac, acceptor splice site; AfSI, AfroSINE insertion; BD, boundary deletion; D, deletion; Do, donor splice site; E, exon; I, insertion; In, intron; INV, inversion; NBR = no BLAST results and possible deletion of exon(s) or gene; NRM = no reads mapped and possible deletion of exon(s) or gene; S, premature stop codon; SCM, start codon mutation; TCM, termination codon mutation; UTR, untranslated region. * Locations of inactivating mutations in exons are based on alignments for complete protein-coding sequences including frameshift insertions when present. # Genes that are pleiotropic rather than eye specific. ^A^ The inversion of exon 2 in *Chrysochloris* may also be present in *Amblysomus*, but the possibility of this inversion was not determined from short reads. ^B^ The only maps to read sequences were from GJA3.

The only evidence for inactivation of a rod phototransduction activation gene in *Notoryctes typhlops* is a GT to AT donor splice site mutation, with 23X coverage, in intron 6 of *PDE6B* that is found in one of the two individuals of *N. typhlops* (Figure S1). In addition to lacking traditional inactivating mutations, the rod opsin gene (*RHO*) has intact critical functional sites and transmembrane domains. Of the three rod phototransduction recovery genes, *SAG* and *SLC24A1* have inactivating mutations in both individuals, whereas *GRK1* is intact in both individuals.

Among the lens/cornea genes, there are shared inactivating mutations in all seven of the crystallin genes that are eye specific (*CRYAA*, *CRYBA1*, *CRYBA4*, *CRYBB1*, *CRYBB2*, *CRYBB3*, *CRYGS*), whereas the pleiotropic *CRYAB* gene remains intact (Table 3). The number of shared inactivating mutations that occur in both individuals of *Notoryctes typhlops* ranges from one in *CRYAA*, *CRYBA4*, and *CRYBB3* to seven in *CRYBA1*. Beyond these shared mutations in various cryatallin genes, additional inactivating mutations occur in one of the two individuals of *N. typhlops* in *CRYBA1*, *CRYBA4*, *CRYBB2*, *CRYBB3*, and *CRYGS*.

Five of six non-crystallin lens/cornea-specific genes (*BFSP1*, *BFSP2*, *GJA8*, *MIP*, *KRT12*) exhibit shared mutations in both individuals of *Notoryctes typhlops*. *LIM2* has an intact coding sequence in *N. typhlops* (but see the next section on golden moles for evidence of inactivation of this gene).

### 3.3. Gene Inactivation in Golden Moles

Inactivating mutations in the cone and rod phototransduction genes of both chrysochlorids are provided in Table 2. Inactivating mutations in the lens/cornea genes of both chrysochlorids are listed in Table 3. Figure 2 provides a visual summary of eye genes that are intact or have inactivating mutations in the two chrysochlorid species. Figure 3 provides examples of inactivating mutations that are found in Chrysochloridae. Among the genes that are specific for cone phototransduction activation, three genes (*CNGB3*, *OPN1LW*, *OPN1SW*) show evidence for inactivation in the common ancestor of the two chrysochlorids (*Amblysomus hottentotus*, *Chrysochloris asiatica*). The shared mutation in *OPN1SW* is a 9 bp deletion in exon 1, which deletes two spectral tuning sites, including the critical site 93, within the second transmembrane region. All eye-specific cone phototransduction activation genes except for *GNGT2* have autapomorphic inactivating mutations in *A. hottentotus* and/or *C. asiatica*. The pleiotropic cone phototransduction activation gene *GNB3* is intact in both chrysochlorids. 

We investigated only two of the three cone phototransduction recovery genes in Chrysochloridae, *GRK7* and *SLC24A2*, because the third recovery gene (*ARR3*) is a pseudogene in Afrotheria [30]. *GRK7* exhibits inactivating mutations that are shared by both chrysochlorids, as well as autapomorphic mutations in both *Amblysomus hottentotus* and *Chrysochloris asiatica* (Table 2). The pleiotropic *SLC24A2* gene is intact in both chrysochlorids.

All of the rod phototransduction activation genes are intact in both chrysochlorids including canonical splice sites (GT/GC donor, AG acceptor) and functional sites in *RHO*. Among rod phototransduction recovery genes, both *GRK1* and *SAG* are intact, whereas *SLC24A1* exhibits a 22 bp (*Amblysomus hottentotus*) or 23 bp (*Chrysochloris asiatica*) frameshift deletion in exon 4 that shares the same 3′ boundary in these two taxa.

Among the 14 lens/cornea genes, the pleiotropic *CRYAB* is intact in both chrysochlorids, whereas all 13 of the eye-specific genes show evidence of pseudogenization in *Amblysomus hottentotus*, *Chrysochloris asiatica*, or both taxa (Table 3).

All six of the non-crystallin lens/cornea genes exhibit inactivating mutations in one or both of the chrysochlorids (Table 3). Genes with inactivating mutations that are shared by *Amblysomus hottentotus* and *Chrysochloris asiatica* include *BFSP1*, *MIP*, and the keratin gene *KRT12*. Autapomorphic inactivating mutations in *A. hottentotus* are found in *BFSP1*, *BFSP2*, *GJA8*, *MIP*, and *KRT12*. *C. asiatica* exhibits autapomorphic inactivating mutations in *BFSP1*, *BFSP2*, *GJA8* (including an AfroSINE insertion), *LIM2*, *MIP*, and *KRT12*.

### 3.4. Gene Inactivation in Other Taxa

Inactivating mutations in taxa other than the two chrysochlorid species and the two individuals of *Notoryctes typhlops* are summarized in Table S2. The diprotodontian marsupial *Strigocuscus gymnotis* (ground cuscus) exhibits inactivating mutations in *OPN1SW*, which encode one of the opsins in the cone phototransduction activation pathway. These mutations render *S. gymnotis*, which is nocturnal, an L-cone monochromat. A second marsupial, *Myrmecobius fasciatus* (numbat), has inactivated copies of two genes in the rod phototransduction recovery pathway, *GRK1* and *SLC24A1*. Among afrotheres, both tenrecs (*Echinops telfairi* (lessser hedgehog tenrec), *Microgale talazaci* (Talazac’s shrew tenrec)) have negative BLAST results for the lens crytallin gene *CRYBA4*. These results suggest that *CRYBA4* is absent or highly degraded in these taxa. One or both tenrecs also present inactivating mutations in one cone phototransduction recovery gene (*GRK7*) and the lens crystallin genes *CRYBB1* and *CRYBB3*.

### 3.5. Selection Analyses and the Timing of Gene Inactivation

To understand the timing of relaxed selection (i.e., commencement of neutral evolution) on eye-specific genes in Chrysochloridae and *Notoryctes typhlops*, we performed selection analyses on groups of genes that are associated with specific functions (cone phototransduction activation, cone phototransduction recovery, rod phototransduction activation (*N. typhlops* only), rod phototransduction recovery, lens/cornea development and structure) and integrated these results with divergence time estimates that were taken from the literature [11,77]. Note that for some (or all) genes, relaxed selection may have commenced before the occurrence of the first inactivating mutation [78]. Mean inactivation times for each group of genes are provided in Table 4 and serve as proxies for relaxed selection on the associated phenotypes. Mean inactivation times are based on eight different combinations of codon frequency model (CF1 or CF2), one versus two synonymous substitution rates, and estimated versus fixed values for the fully pseudogenic dN/dS category [3]. Individual inactivation time estimates for each of these eight combinations are provided in Table S3.

**Figure 4:**
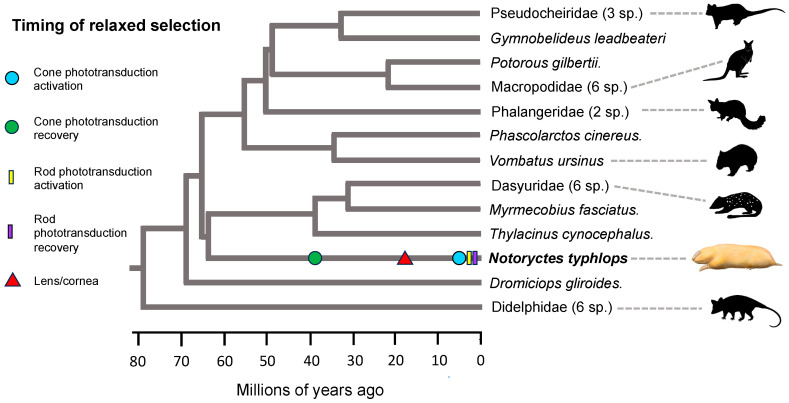
Estimated times for the commencement of relaxed selection (neutral evolution) in five groups of eye genes in *Notoryctes typhlops*. Mean estimates are the mean of eight individual estimates for each gene group (Table S2). Divergence times for Pseudocheiridae to *Gymnobelideus leadbeateri* are from Mitchell et al. [86]. All other divergence dates are from Duchêne et al. [77]. Silhouettes are for the same species or a closely related species.

**Figure 5:**
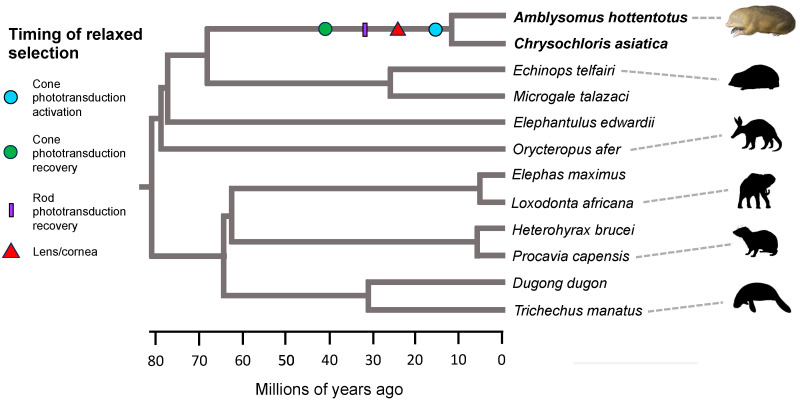
Estimated times for the commencement of relaxed selection (neutral evolution) in four groups of eye genes in Chrysochloridae. Mean estimates are the mean of eight individual estimates for each gene group (Table S2). The divergence time for *Echinops telfairi* to *Microgale talazaci* is from Foley et al. [74]. All other divergence times are from Meredith et al. [11]. Silhouettes are for the same species or a closely related species.

Estimated dates for the commencement of relaxed selection on different groups of eye-specific genes are summarized in Table 4, Figure 4 (*Notoryctes typhlops*), and Figure 5 (chrysochlorids). Mean estimates for relaxed selection on cone phototransduction activation genes in Chrysochloridae and *N. typhlops* are ~16.21 and ~5.38 MYA, respectively. In the case of *N. typhlops*, the dN/dS values for the fully pseudogenic category are ~2.32 (CF1) and ~2.35 (CF2) and are suggestive of positive selection, but these values are based on a small number of substitutions (7.1–7.2) in this category and are not significantly different from a neutral value of 1.0. For cone phototransduction recovery genes (one in Afrotheria, two in Marsupialia), mean estimates for relaxed selection times are ~40.31 for Chrysochloridae and ~38.85 MYA for *N. typhlops*. We also estimated a relaxed selection time for rod phototransduction activation (six genes after excluding *CNGB1*, *GNB1*, *RHO*) in *N. typhlops* given the absence of rods in this taxon [16] and evidence for the inactivation of *PDE6B* in one of the two individuals. Our estimate for relaxed selection on rod phototransduction activation in *N. typhlops* is ~3.39 MYA. The estimated dN/dS values for the fully pseudogenic category are elevated above neutral expectations (~1.93 (CF1), ~1.98 (CF2)), but as for the cone phototransduction activation genes these values are based on a small number of substitutions (12.2–12.6) in this category and are not significantly different from a neutral value of 1.0 based on ln likelihood ratio tests (Table S3). Nevertheless, selection models with three categories for dN/dS values (background, stem *Notoryctes*, crown *Notoryctes*) are significantly better than models with a single dN/dS category (Table S3). For three rod phototransduction recovery genes, estimates for the onset of relaxed selection are ~31.43 MYA for Chrysochloridae and ~1.52 MYA for *N. typhlops*. Finally, estimates for relaxed selection on lens/cornea genes (12 genes in Afrotheria, 13 genes in Marsupialia) are ~26.03 MYA for Chrysochloridae and ~17.84 MYA for *N. typhlops*.

## 4. Discussion

### 4.1. Patterns of Inactivation in the Eye Genes of Golden Moles and Marsupial Moles

We examined 12 cone phototransduction genes, 12 rod phototransduction genes, and 14 lens/cornea genes in 28 marsupial and 12 afrothere species. With six exceptions (*CNGB1*, *CRYAB*, *GNB1*, *GNB3*, *RHO* [=*OPN1*], *SLC24A2*), these genes are hypothesized to be eye-specific, in which case we should expect to find inactivating mutations in some or all of these genes in *Notoryctes typhlops* and the two chrysochlorids, given that these taxa have degenerate eyes that are covered by skin and fur and lack an optic nerve connection between the eyes and the brain. 

Among genes that are specific to cone phototransduction, the two individuals of *Notoryctes typhlops* exhibit shared inactivating mutations in three cone phototransduction activation genes (*CNGA3*, *CNGB3*, *GNAT2*) and one cone phototransduction recovery gene (*ARR3*). A second cone phototransduction recovery gene (*GRK7*) is inactivated in the DNA Zoo individual of *N. typhlops*. The total number of inactivating mutations in these cone phototransduction genes is 12 in the NYGC individual (7 inactivation + 5 recovery) and 12 in the DNA Zoo individual (6 activation + 6 recovery). In contrast with *N. typhlops*, both chrysochlorids have more inactivated cone phototransduction genes (5 in *Amblysomus hottentotus*, 7 in *Chrysochloris asiatica*) and more inactivating mutations in these genes (35 in *A. hottentotus*, 45 in *C. asiatica*) even though there is one-less cone-specific gene in Afrotheria than Marsupialia (*ARR3* is a pseudogene in all afrotherians). These results are in striking contrast to Sweet’s [16,17] morphological studies of the degenerate eyes of *N. typhlops* and two chrysochlorids (*A. hottentotus*, *C. asiatica*). Specifically, rods and cones are both absent in *N. typhlops* [17], whereas “the layer of rods and cones is more or less distinct” in chrysochlorids [16], p. 335. Thus, we may have expected more inactivating mutations in *N. typhlops* than in chrysochlorids if morphological degeneration of the eyes and the molecular decay of cone phototransduction genes proceeded in synchrony.

In the case of lens/cornea-specific genes, both *Notoryctes typhlops* individuals have 12 genes with inactivating mutations, and the total number of inactivating mutations is 49 for the NYGC animal and 46 for the DNA Zoo animal. The mean number of inactivated lens/cornea genes is also 12 for the two chrysochlorids (11 in *Amblysomus hottentotus*, 13 in *Chrysochloris asiatica*), but these taxa exhibit more inactivating mutations in their pseudogenic lens/cornea genes than are present in *N. typhlops*, i.e., 55 inactivating mutations in *A. hottentotus* (26 shared + 29 autapomorphic) and 79 inactivating mutations in *C. asiatica* (26 shared + 53 autapomorphic). *C. asiatica* exhibits at least one inactivating mutation in 13 of 13 lens/cornea-specific genes. The only lens/cornea-specific gene that lacks inactivating mutations in *N. typhlops* is *LIM2*, and the only lens/cornea-specific genes that lack inactivating mutations in *A. hottentotus* are *CRYAA* and *LIM2*. As for the cone phototransduction genes, the greater extent of molecular decay in chrysochlorids than in *N. typhlops* is surprising given that the lens is degenerate, although recognizable, in chrysochlorids [16], whereas the lens is absent in *N. typhlops* [17,18].

In summary, both cone phototransduction and lens/cornea genes exhibit more inactivating mutations in chrysochlorids (35 + 55 = 90 in *Amblysomus hottentotus*, 45 + 79 = 124 in *Chrysochloris asiatica*) than in *Notoryctes typhlops* (49 +12 = 61 in NYGC individual, 46 + 12 = 58 in DNA Zoo individual) even though the cones and remnants of the lens are both present in chrysochlorids but not *N. typhlops*. One explanation for these discordant patterns is that the tempo of morphological degeneration was impacted more strongly by natural selection in *N. typhlops* than in chrysochlorids [17]. Indeed, Sweet [17] hypothesized that vestigialization of the eyes occurred rapidly and recently in *N. typhlops* to prevent injuries to the eyes from grains of sand and to make room for enlarged glands that secrete material to protect the nasal passages from the same sand. On the other hand, at the molecular level the vast majority of inactivating mutations in eye-specific genes or groups of eye-specific genes are expected to be neutral even if the initial inactivating mutation in a gene was favored by natural selection. This is because the initial inactivating mutation may have eliminated a particular structure (e.g., functional lens) or physiological process (e.g., cone phototransduction) in the eye, whereas subsequent mutations would generally be redundant rather than additive. For example, a single mutation in either *CNGA3* or *CNGB3* in the cone phototransduction activation pathway can cause achromatopsia (=rod monochromacy) and eliminate photopic vision [80]. If the first inactivating mutation occurred in *CNGA3*, then subsequent mutations in this gene or in other eye-specific genes in the cone phototransduction activation pathway will simply be redundant since the first mutation would have already eliminated cone vision. Similarly, single mutations in one of the lens-forming genes (humans or mice) can result in a misshapen and/or opaque lens, often with cataracts, that does not refract or transmit light properly for the purpose of image formation [81,82,83]. Subsequent mutations will simply be redundant for loss of an image-forming lens. Thus, the accumulation of additional inactivating mutations should mostly follow a neutral trajectory even if the first mutation was favored by natural selection. An exception may be cases in which the initial inactivating mutation is located in the 3’ region of the coding sequence so that there is still a cost associated with the production of an unnecessary and potentially non-functional protein, whereas an upstream mutation may eliminate or greatly reduce this cost. Also, the rapid evolution of small, vestigial eyes may have more to do with regulatory genes, such as transcription factors, that affect rates of development and the timing of onset/offset for the growth of different features of an organism. Along these lines, genes such as *PAX6*, *LHX2*, *SIX6*, and *RAX* are known to be important for proper development of the eye [84], and mutations in these genes may turn out to be important in the vestigialization or elimination of different components of the eye in moles. Microphthalmia (small eyes) may be a simple failure of the eye vesicle to grow at a sufficient pace at the appropriate embryonic stages [21,85]. Gubbay [21] compared the rate of development of the optic vesicle/cup in Grant’s golden mole (*Eremitalpa granti*) to that of an elephant shrew (*Elephantulus myurus*) and found that the optic cup of *Er. granti* increased by a factor of 2.5, whereas the eye cup of *El. myurus* increased by a factor of 15.6 during the same time (stage) interval.

In contrast with the extensive evidence for pseudogenization of cone phototransduction and lens/cornea genes in both *Notoryctes typhlops* and chrysochlorids, there is limited evidence for the pseudogenization of rod phototransduction activation genes in *N. typhlops* and no evidence for pseudogenization of these genes in chrysochlorids. In the case of *Notoryctes typhlops*, every rod phototransduction activation gene is intact in the DNA Zoo individual, and the only potential inactivating mutation in the NYGC individual is a splice site mutation in intron 6 of the *PDE6B* gene. This gene encodes one of the two catalytic subunits of rod phosphodiesterase (PDE). Mutations in this gene are associated with retinitis pigmentosa in humans and mice [87,88]. Two isoforms of PDE6B are known, but neither isoform involves alternative splicing associated with intron 6 [89]. Perhaps the polymorphic mutation in *N. typhlops PDE6B,* represents the first step in the degradation of the rod phototransduction activation pathway. Even so, it is surprising that the rod phototransduction activation pathway is either intact (DNA Zoo individual) or in a very early stage of decay (NYGC individual) when rods are completely absent and there is no optic nerve connection to the brain [17].

Both chrysochlorids, in turn, have an intact set of rod phototransduction activation genes with canonical splice sites, and there is no evidence for inactivation of the rod phototransduction activation pathway. As for *N. typhlops*, this is perplexing given that the optic nerve is also severed in several chrysochlorid species that have been examined [16,20,22].

Why is the rod phototransduction activation pathway still intact in both chrysochlorids and one of the two individuals of *Notoryctes typhlops* if the optic nerve connection between the eye and the brain has been eliminated in both taxa? We propose several hypotheses to account for this conundrum. First, early reports of a severed optic nerve connection between the eye and the brain in *N. typhlops* [17] and chrysochlorids [16] may have been an artefact of preparation techniques that were used at that time. However, such artefacts do not seem to have impacted contemporaneous studies of the eye in other mammalian taxa including small-bodied moles and shrews [90,91]. Also, more-recent work has confirmed the absence of an optic nerve exiting the brain in several species of chrysochlorids [reviewed in 21]. Second, the absence of optic nerve connections between the eyes and the brain may have occurred recently in both *N. typhlops* and different chrysochlorid species, in which case rod-specific phototransduction genes have had very little time to accumulate inactivating mutations. However, it is more parsimonious to assume that the optic nerve connection was lost on the stem chrysochlorid branch given that multiple chrysochlorid species, including species (i.e., *Chrysochloris asiatica*, *Amblysomus hottentotus*) that may index the basal split in Chrysochloridae [27,28], exhibit this condition. Third, most or all rod phototransduction activation genes may be pleiotropic and have critical roles in extraocular tissues. Pleiotropic roles are known for *CNGB1* in the olfactory cascade, *GNB1* in brain neurons [92,93], and *RHO* in the thermotaxis of mammalian sperm [51,52]. A role of *RHO* in sperm thermotaxis is of particular interest because a variety of opsin proteins (OPN2, OPN3, OPN4, OPN5) are now implicated as thermal sensors in different mammalian tissues [94]. If most or all rod phototransduction activation proteins are pleiotropic, possibly in connection with the sperm thermotaxis that begins with rhodopsin, they would not be expected to accumulate inactivating mutations in blind notoryctids or chrysochlorids. However, the precise identity of the downstream players in the transducin/cyclic nucleotide signaling cascade that begins with rhodopsin remain to be identified [52]. Most rod phototransduction activation genes (*CNGA1*, *CNGB1*, *PDE6A*, *PDE6B*, *PDE6G*, *RHO*) are associated with non-syndromic retinitis pigmentosa rather than syndromic retinitis pigmentosa [95], in which case pleiotropic roles may be unexpected. As noted above, however, two of these retinitis pigmentosa genes (*CNGB1*, *RHO*) have pleiotropic roles in extraocular tissues. The sense of smell and sperm thermotaxis may have been compromised by mutations in *CNGB1* and *RHO*, respectively, but screens that are used to classify retinitis pigmentosa mutations as non-syndromic versus syndromic are likely to have missed the potentially detrimental effects of these mutations to the affected physiological processes. One of the rod phototransduction activation genes that may have a role in extraocular tissues is *GNAT1*. This gene is expressed at low levels in rat taste cells [96] and also in human testis and sperm [97]. However, the physiological and fitness consequences of mutations in this gene in extraocular tissues are unclear. The main G protein alpha subunit in gustducin is *GNAT3*, not *GNAT1*, and the physiological significance of the low-level expression of the latter in rat taste cells remains to be investigated.

Unlike genes in the rod phototransduction activation pathway, *Notoryctes typhlops* and both chrysochlorids exhibit molecular evidence for degradation of the rod phototransduction recovery pathway. The DNA Zoo individual of *N. typhlops* exhibits ten inactivating mutations that are spread across two rod phototransduction recovery genes (*SAG* (four mutations), *SLC24A1* (six mutations)). The NYGC animal has an additional mutation in each of these genes. Both chrysochlorids have a single inactivating mutation in the *SLC24A1* gene. The greater degeneration of rod phototransduction recovery genes in *N. typhlops* than chrysochlorids parallels the greater degeneration of rods in *N. typhlops* than chrysochlorids. More specifically, rods are absent in *N. typhlops* [16] but still present in chrysochlorids [16]. Again, this difference may be moot given the severed optic nerve in *N. typhlops* and both chrysochlorid species [16,17]. *SLC24A1* and a second rod phototransduction recovery gene (*GRK1*) also have inactivating mutations in the numbat, which is a diurnal marsupial. In addition, Emerling and Springer [30] reported negative BLAST results for *SLC24A1* in *Condylura cristata* (star-nosed mole). So, the loss of rod recovery genes is not unprecedented in mammals even if we ignore golden moles and chrysochlorids. Vinberg et al. [98] found that *SLC24A1*-deficient mice did not experience severe degeneration of their rods and suggested that other mechanisms may exist for Ca^++^ extrusion from the outer segments of rods when *SLC24A1* is ablated.

### 4.2. The Tempo of Convergent Eye Degeneration in Golden Moles and Marsupial Moles

Chrysochlorids and *Notoryctes typhlops* have the most degenerate eyes in all of Mammalia, and the eyes of the latter may be more degraded than those of any other terrestrial vertebrate [16,17]. Chrysochlorids and *N. typhlops* both have eyes that are covered by skin and fur, lack or have highly degenerate extrinsic ocular muscles, lack trochlear and oculomotor nerves that supply innervation to five of the six extrinsic muscles, lack a lens or have a degenerate lens, lack an iris or have a degenerate iris, and lack optic nerve connections between the eyes and the brain (Table 1). In addition, rods and cones are no longer present in *N. typhlops*. Sweet [17] hypothesized that the eyes of the marsupial mole were rapidly and recently vestigialized, under the influence of natural selection and in response to the aridification of Australia with the emergence of sandy deserts, to (1) mitigate against the injurious effects of getting sand in the eyes and (2) accommodate the enlarged glands and their secretions that keep the nasal cavity moist and prevent sand from entering the nasal passages during burrowing. If Sweet’s hypothesis is correct, there may have been less time for the accumulation of inactivating mutations in the eye-specific genes of *N. typhlops* than in chrysochlorids, even though the former has more degenerate eyes. Here, we present data and analyses that provide some support for Sweet’s [17] hypothesis for the accelerated degeneration of the eyes in *N. typhlops* relative to the most comparable group in Placentalia—Chrysochloridae.

We estimate that selection was relaxed on cone phototransduction recovery genes ~38.9 MYA, lens/cornea genes ~17.84 MYA, and cone phototransduction activation genes ~5.38 MYA in *Notoryctes typhlops*. Estimates for relaxed selection in chrysochlorids are older for each set of genes, but are in the same temporal sequence: cone phototransduction recovery genes ~40.31 MYA, lens/cornea genes ~26.03 MYA, and cone phototransduction activation genes ~16.21 MYA. For cone phototransduction, selection on the recovery gene(s) was relaxed much earlier than selection on activation genes in both *N. typhlops* and chrysochlorids. The inactivated cone phototransduction recovery genes in *N. typyhlops* are *ARR3* and *GRK7*. In chrysochlorids, the inactivated cone phototransduction gene is *GRK7* (*ARR3* is a pseudogene in all afrotherians). The significance of these losses for cone vision is somewhat unclear given the possibility of redundancies in the cone phototransduction recovery system. As noted by Emerling and Springer [30], the inactivation of *ARR3* in afrotherians may have been rescued by *SAG*, which is highly expressed in mouse cones [99]. The same suggestion applies to the loss of *ARR3* in *N. typhlops* if *ARR3* was inactivated before *SAG*. In the case of *GRK7*, which is inactivated in *N. typhlops* and chrysochlorids, this loss may have been rescued by *GRK1*, which remains intact in both taxa. Thus, relatively early dates for relaxed selection on cone recovery genes do not preclude a version of cone phototransduction recovery that made use of redundant genes from the rod recovery pathway.

Lens/cornea genes were the next set of genes to experience relaxed selection in *Notoryctes typhlops* and chrysochlorids. Crystallin genes and other lens/cornea genes encode proteins that perform a variety of functions that are essential for proper development of the lens and cornea. Alpha, beta, and gamma crystallins make critical contributions to the transparency and refractive index of the lens. Crystallins can also interact with other lens proteins. For example, CRYAA forms complexes with the beaded-filament structural proteins 1 (filensin) and 2 (phakinin) of the lens that are encoded by the *BFSP1* and *BFSP2* genes [100]. *KRT12*, in turn, encodes a cornea-specific keratin protein. *CRYBA4* and *CRYBB1* also encode for proteins that affect the size of the cornea [53]. Our estimates for the onset of relaxed selection (neutral evolution) in the lens/cornea genes of both *N. typhlops* and chrysochlorids, one of which is in the Miocene (*N. typhlops*) and the other in the Oligocene (chrysochlorids), are consistent with the ancient accumulation of numerous inactivating mutations across many genes (Table 3). However, it is striking that the lens of chrysochlorids is still recognizable after ~26.03 MY of neutral evolution, whereas there is no trace of a lens in *N. typhlops* after ~17.84 MY of neutral evolution. In both *N. typhlops* and chrysochlorids, the cornea is indistinguishable from the choroid and sclera [16,17].

The onset of relaxed selection on cone phototransduction activation genes occurred more recently than did the onset of relaxed selection on cone phototransduction recovery genes or lens/cornea genes. Potential reasons for the early onset of relaxed selection on cone recovery genes have already been discussed. In the case of lens/cornea genes versus cone activation genes, precise image formation may have been less important than other aspects of vision such as color discrimination or simple light detection when visual acuity was waning. Relaxed selection on cone phototransduction activation genes in *Notoryctes typhlops* dates to the earliest Pliocene (~5.38 MYA), whereas the onset of neutral evolution in the same genes in chrysochlorids commenced more than ten MY earlier (~16.21 MYA). As for lens/cornea genes, the difference between these dates is notable given that cones are still recognizable in chrysochlorids, whereas they are absent in *N. typhlops.*

### 4.3. Notoryctid Evolution and the Fossil Record

With the exception of an isolated tooth from the late Oligocene [101], the oldest notoryctid fossil is *Naraboryctes philcreaseri* from the early Miocene of Riversleigh, Queensland [101,102]. The Miocene deposits from Riversleigh have been interpreted as a closed rainforest habitat. *Na. philcreaseri* is known from both cranial and postcranial material. The postcranial skeleton, which includes elements from both the front and hind limb, exhibits adaptations for fossoriality that are similar to, but not as well developed as, in *Notoryctes* [101,102]. Archer et al. [102] suggested that *Naraboryctes* was less specialized for eating soft-bodied invertebrates than *Notoryctes* and may have spent less time feeding underground. Importantly, the paleoenvironment and anatomical features of *Naraboryctes* suggest that the first steps in the evolution of fossoriality in notoryctids did not occur in the arid, sandy deserts of Australia but instead on soft rainforest floors [101,102]. Our selection analyses suggest that lens/cornea-specific genes began evolving neutrally ~17.84 MYA in the Miocene. At this time, at least one notoryctid species was burrowing in the soft soils of Australian rainforests.

Archer et al. [102], p. 1503 further suggested that Miocene notoryctids were “serendipitously preadapted in terms of strategies for avoiding physiological stresses, to the drier environments that developed in central Australia from the late Miocene onwards as rainforests gradually retreated to coastal margins…”. Grasses become more abundant in Australia in the late Miocene at ~6 MYA, and grasslands became widespread during the middle Pliocene [101,103]. Beck et al. [101] suggested that the aridification of Australia caused notoryctids to spend more time underground because they were more vulnerable to predators in open environments. The initiation of continental dune fields in Central Australia commenced as recently as ~1 MYA [104], but prior to the dune fields there were stable sand plains [105]. Coastal sand plains in southwestern Australia may trace as far back as the latest Miocene ~5.3 MYA [26,106]. The ablation of cone phototransduction activation coincides with the aridification of Australia. Our estimate for the onset of neutral evolution in cone phototransduction activation genes is ~5.38 MYA in the latest Miocene. This estimate coincides with evidence for the first coastal sand plains in Australia. The timing of neutral evolution for the complete set of rod phototransduction genes is even smaller (~3.39 MYA), but an important caveat here is that only one rod activation gene (*PDE6B*) is pseudogenized in *Notoryctes typhlops,* and the single inactivating mutation occurs in just one of the two sequenced individuals. Neutral evolution on the rod phototransduction activation genes of *Notoryctes* may be in a very early stage, but it is also possible that some or most genes in the rod phototransduction activation cascade are pleiotropic and are expressed in extraocular tissues.

### 4.4. Chrysochlorid Evolution and the Fossil Record

The oldest putative chrysochlorid fossils are *Diamantochloris inconcessus* and *Damarachloris primaevus* from the middle Eocene (Lutetian) Black Crow limestone deposits of Namibia [107,108]. *Di. inconcessus* is known from a single lower molar, and *Da. primaevus* is known from a maxilla with three teeth and two lower molars. As such, these fossils provide very limited information on the evolution of fossoriality in Chrysochloridae. Pickford [109] also described the chrysochlorid fossil *Namachloris arenatans* from EoCliff in Namibia. Pickford [109] suggested a Bartonian age (41.2–37.71 MYA) for the EoCliff deposits; although, an Oligocene age seems more likely [110,111]. Whatever the correct age, *N. arenatans* is the most anatomically complete pre-Pliocene golden mole [111] and is known from extensive skeletal material including cranial and postcranial elements that bear directly on the evolution of fossoriality in Chrysochloridae [111]. Anatomical features of the postcranial anatomy of this species include a greatly elongated medial epicondyle of the humerus and suggest that *N. arenatans* was fossorial and may have been a “sand swimmer” like the extant *Eremitalpa granti* [109,111]. *N. arenatans* also has some of the derived features of the inner-ear and middle-ear cavity that are found in extant chrysochlorids including a highly coiled cochlea and a pneumatized, trabeculated basicranium, and lateral skull wall [110]. These features are thought to be related to the enhanced low-frequency hearing of chrysochlorids in subterranean environments [110,112] and provide additional evidence for well-developed fossoriality in *N. arenatans*. Given the tendency of fossorial taxa to reduce or eliminate their eyes [30,112,113], the greater antiquity of the oldest chrysochlorid fossil with fossorial adaptations (*Namachloris arenatans*) than the oldest notoryctid fossil with fossorial adaptations (*Naraboryctes philcreaseri*) is consistent with molecular evidence for the earlier onset of eye degeneration in chrysochlorids than notoryctids (~26.03 versus ~17.84 MYA for lens/cornea genes; ~16.21 versus ~5.38 MYA for cone phototransduction activation genes). A caveat is that the fossil record is very sparse for these taxa so that future discoveries of extinct species may alter our understanding of when fossoriality evolved in chrysochlorids and notoryctids.

### 4.5. Circadian Photoentrainment in Golden Moles and Marsupial Moles

Nearly all mammalian tissues have autonomous circadian clocks that must be entrained to the 24 h day [114]. In mammals and most other organisms, the primary external cues that enable entrainment (=zeitgeber) are changes in the quality and quantity of light at dawn and dusk [115]. Mammalian photoentrainment relies on direct input from the eyes to the suprachiasmatic nucleus, which is the master clock and principal pacemaker for circadian rhythms [116]. Within the eye, photosensitive cells that convey signals to the suprachiasmatic nucleus include intrinsically photosensitive retinal ganglion cells (ipRGCs), rods, and cones [117]. The primary G protein-coupled receptor in ipRGCs is melanopsin, which is encoded by the *OPN4* gene. Melanopsin-containing ipRGCs play a major role in photoentrainment and provide information regarding bright light over extended periods of time [115]. Rods and cones, in turn, communicate with the ipRGCs through intermediate neurons and modify the endogenous light responses of the ipRGCs [115]. The input of rods is important because ipRGCs are incapable of driving physiological responses at low light levels [117,118]. Cones are important for the detection of light at higher intensities and for the integration of intermittent light exposure [115].

The Iberian Mole (*Talpa occidentalis*) and the Middle East blind mole rat (*Nannospalax ehrenbergi*) are similar to marsupial moles and golden moles in having vestigial eyes that are covered by skin. *T. occidentalis* exhibits lens abnormalities, but the retina is well conserved and there are melanopsin-containing ipRGCs. Carmona et al. [31] concluded that the Iberian mole’s visual functions are probably limited to sunlight detection and the maintenance of circadian rhythms. In *Na. ehrenbergi*, the eyes have regressed further and are not sensitive to light. However, they still play a role in circadian entrainment [119]. The retinal ganglion layer is still present in *Na. ehrenbergi,* and there is a very small unmyelinated optic nerve comprised of ~900–1000 fibers [120]. In contrast, the eyes of *Notoryctes* and Chrysochloridae have degenerated beyond this condition and there is no optic nerve to connect the retinal ganglion layer to the suprachiasmatic nucleus in the brain. Given the importance of the eye for circadian entrainment in other mammals, including the subterranean *T. occidentalis* and *Na. ehrenbergi*, how does circadian entrainment occur in *Notoryctes* and Chrysochloridae when there are no optic nerve connections between the eyes and the brain? And in the case of *Notoryctes* when rods, cones, and the ganglion layer of the retina are all absent? One hypothesis is that these taxa rely on temperature for circadian entrainment. The effects of 24 h temperature cycles on circadian entrainment have only been studied in a handful of mammalian species, primarily rodents, primates, and bats. One takeaway from these studies is that homeothermic species are less likely to be temperature entrained than heterothermic species. For example, six of twelve individuals of the heterothermic *Molosssus rufus* (=*M. ater*, black mastiff bat) were entrained by cycles of 20 °C to 30 °C, whereas zero of four individuals of the homeothermic *Phyllostomus discolor* (pale spear-nosed bat) were entrained with the same temperature treatment [121]. A second takeaway is that temperature entrainment, when successful, only affects some of the tested animals [120,122,123]. For example, Goldman et al. [120] found that half of the individuals of *N. ehrenbergi* that were tested showed entrainment to daily cycles of ambient temperature. Similarly, Aschofff and Tokura [123] found that exposure to cycling ambient temperature resulted in entrainment in 45% of the trials with squirrel monkeys (*Saimiri sciurus*). The general conclusion from these studies is that light is a more effective zeitgeber than temperature for circadian entrainment [120]. Nevertheless, temperature may provide a sufficient entrainment cue for subterranean mammals such as chrysochlorids and *Notoryctes* that spend most of their lives underground, especially because these mammals are heterothermic [124,125].

A second hypothesis is that some form of photoentrainment is facilitated by the photopigment neuropsin, which is encoded by the *OPN5* gene. Buhr et al. [114] suggested this hypothesis based on their work with cultures of murine outer ear and vibrissal skin, which demonstrated that photoentrainment of these cultures was mediated by the *OPN5* gene in the presence of an exogenous source of retinaldehyde. These results challenge the conventional view that peripheral circadian rhythms in mammals are synchronized exclusively by the suprachiasmatic nucleus via ocular photoreception, and further suggest that non-visual light sensing in peripheral tissues is also important for this purpose [114]. We note that chrysochlorids (*Amblysomus hottentotus, Chrysochloris asiatica*) and *Notoryctes typhlops* have intact *OPN5* coding sequences including canonical splice sites. Together, entrainment to daily temperature cycles and OPN5-mediated photoentrainment in the skin may provide sufficient information for the circadian rhythms of chrysochlorids and *Notoryctes*.

## 5. Conclusions

Chrysochlorids and *Notoryctes* have the most degenerate eyes of any mammalian taxa. Of these, the eyes of *Notoryctes* are perhaps more degenerate than those of any other terrestrial vertebrate. If the eyes of chrysochlorids and *Notoryctes* were vestigialized at the same rate under relaxed selection, then the transition from purifying selection to neutral evolution on eye-specific genes should have occurred earlier in *Notoryctes* than in chrysochlorids because the former has more degenerate eyes than the latter. An alternate hypothesis is that degeneration of the eyes occurred rapidly and recently in *Notoryctes* in response to the aridification of Australia and the formation of sandy deserts that *Notoryctes* inhabits [17]. To test these hypotheses, we examined eye-specific genes that are associated with cone phototransduction, rod phototransduction, and development of the lens/cornea in 12 afrotherians and 28 marsupials. The two chrysochlorids exhibit more inactivating mutations in the cone phototransduction genes and lens/cornea genes than does *Notoryctes typhlops*. Our estimates for the onset of relaxed selection on eye-specific genes in these categories are also older for chrysochlorids than for the *N. typhlops* lineage. For both taxa, the onset of relaxed selection followed the same temporal sequence. First, cone phototransduction recovery genes: 40.31 MYA (Chrysochloridae) versus 38.85 MYA (*N. typhlops*). Second, lens/cornea genes: 26.03 MYA (Chrysochloridae) versus 17.84 MYA (*N. typhlops*). Finally, cone phototransduction activation genes: 16.21 MYA (Chrysochloridae) versus 5.38 MYA (*N. typhlops*). For the notoryctid lineage, onset of relaxed selection on the lens/cornea genes occurred before the aridification of Australia that commenced in the latest Miocene. The timing of relaxed selection on cone phototransduction activation genes, in turn, coincides with the beginning of Australia’s aridification including evidence for the first coastal sand plains. Overall, these results lend some support to Sweet’s [17] hypothesis for the relatively recent and rapid degeneration of the eyes in *Notoryctes*.

We also observed evidence for pseudogenization of rod phototransduction recovery genes in *N. typhlops* (two genes), both chrysochlorids (one gene), and the diurnal marsupial numbat (two genes). It is possible that the rod recovery pathway, or at least parts thereof, can be rescued by other genes [98]. The rod phototransduction activation pathway, in turn, is intact in both chrysochlorids and one of the two individuals of *N. typhlops*. The second individual of *N. typhlops* has a single inactivating mutation in *PDE6B* that may signal an early stage of degeneration in this gene cascade.

The integrated results of morphological and molecular studies on the eyes and eye genes of chrysochlorids and *Notoryctes* have obvious shortcomings and present unanswered questions that will require additional research. One shortcoming is that much of the previous anatomical work on chrysochlorid and in particular notoryctid eyes is more than a century old. Studies with modern techniques such as confocal microscopy and immunohistochemistry for gene expression should provide a host of new insights into the evolution of the eyes in chrysochlorids and *Notoryctes*, especially if early and late developmental stages can be examined. For example, are any of the intact genes in the rod phototransduction activation cascade, especially *RHO*, still expressed in the eyes of chrysochlorids and *Notoryctes* even though there is no optic nerve connection to the brain in these mammals? If these genes are not expressed in the eye this will reinforce the conclusion that they no longer have a role in light reception in this degenerate organ. On the other hand, if they are expressed in the eye then what is the purpose of this expression? Also, are melatonin-synthesis genes still expressed in the retina? Outside of the eye, which rod phototransduction activation genes are pleiotropic with an additional function(s) (e.g., sperm thermotaxis)? *CNGB1*, *GNB1*, and *RHO* are pleiotropic, based on available evidence [46,47,52], but the possibility of pleiotropic roles for other genes in the rod activation cascade is less clear. It is generally assumed that many of these genes are rod-specific, but if this were the case, we might expect to see inactivating mutations in the rod activation genes of both *Notoryctes* and chrysochlorids given the absence of rods in *Notoryctes* and the absence of an optic nerve connection between the eyes and the brain in both *Notoryctes* and chrysochlorids. Also, what roles have transcription factors, such as *PAX6,* played in the degeneration of the eyes in *Notoryctes* and chrysochlorids? Finally, to what extent does circadian entrainment occur in *Notoryctes* and chrysochlorids, and how is entrainment achieved? Studies that address these questions, and additionally make comparisons to other blind or nearly blind mammals with genomic and anatomical data (e.g., Spalacinae (blind mole-rats), *Fukomys damarensis* (Damara mole-rat), *Heterocephalus glaber* (naked mole-rat)), have the potential to provide key insights into the biology of subterranean mammals, phototransduction, and the circadian clock.

## Figures and Tables

**Table 1:** A comparison of features of the degenerate eyes of chrysochlorids and *Notoryctes typhlops* ^A^.

Feature	Chrysochloridae	*Notoryctes typhlops*
Location of eyes	Not visible externally; located within the dermis and covered by outer layers of skin and fur	Not visible externally; located beneath the temporalis muscle and covered by skin and fur
2. Extrinsic eye muscles	Absent	Degenerative, abnormal in position, non-striated
3. Oculomotor (III), trochlear (IV), and abducens (VI) nerves	III and IV absent; no information on VI	Absent
4. Oculomotor, trochlear, and abducens nuclei in brain	Oculomotor and trochlear nuclei greatly reduced and neurons lack clear dendrites; abducens nucleus absent	No information
5. Optic nerve (II) and optic chiasma	Optic nerve and optic chiasma absent except for remnants of nerve exiting eye in some specimens	Optic nerve and optic chiasma absent except for remnants of nerve or nerve sheath exiting eye in one specimen
6. Iris	Degenerate but recognizable	Absent or represented by only a few nuclei
7. Pupil	Small space that may be pupil	Absent
8. Cornea	Indistinguishable from sclera and choroid	Indistinguishable from sclera and choroid
9. Lens	Degenerate but recognizable; adult lens is an irregular mass of cells without lens fibers	Absent
10. Vitreous body	Not well developed or absent	Practically absent or absent
11. Retina	Some degeneration but layers are clearly distinguishable in most cases	Undifferentiated mass of cells
12. Retinal pigment layer	Well developed	Eye is hollow ball of pigment that may be “greatly changed” retinal pigment layer
13. Ganglion layer in retina	Most degenerate layer of retina	Absent
14. Cones and rods	Layer of cones and rods is recognizable in some, but in other cases all that remains are rod-like structures	Absent

^A^ Features of chrysochlorid eyes are based on Sweet [16], Clark [20], Gubbay [21], Stephan et al. [22], Perrin and Fieldin [23], and Bhadwandin et al. [19] Features of *Notoryctes typhlops* eyes are based on Sweet [17], and Johnson and Walton [18].

**Table 4:** Estimated times in millions of years for the commencement of neutral evolution on groups of eye-specific genes, and by proxy the associated phenotypes, in Chrysochloridae and *Notoryctes typhlops*. Estimates are mean values based on eight different analyses. See Table S2 for estimates based on individual analyses.

Eye Phenotype and Associated Genes	Inactivation Time in Chrysochloridae	Inactivation Time in *Notoryctes typhlops*
Cone phototransduction activation (*CNGA3*, *CNGB3*, *GNAT2*, *GNGT2*, *OPN1LW*, *OPN1SW*, *PDE6C*, *PDE6H*)	16.21	5.38
Cone phototransduction recovery (ARR3 *, GRK7)	40.31	38.85
Rod phototransduction activation (*CNGA1*, *GNAT1*, *GNGT1*, *PDE6A*, *PDE6B*, *PDE6G*)	No inactivated genes	3.39
Rod phototransduction recovery (*GRK1*, *SAG*, *SLC24A1*)	31.43	1.52
Lens/cornea development and structure (*CRYAA*, *CRYBA1*, *CRYBA4 ^#^*, *CRYBB1*, *CRYBB2*, *CRYBB3*, *CRYGS*, *BFSP1*, *BFSP2*, *GJA8*, *LIM2*, *MIP*, *KRT12*)	26.03	17.84

* *ARR3* is a pseudogene in Afrotheria and was not included in the inactivation dating analyses for this taxon. ^#^ *CRYBA4* was not included in the analyses for Afrotheria because there were no BLAST results for this gene in Chrysochloridae.

## Data Availability

Please see Supplementary Materials link (above).

## Supplementary Materials

The following supporting information can be downloaded at: https://www.mdpi.com/article/10.3390/genes14112018/s1. (1) Alignments for protein-coding sequences (CDS) for individual phototransduction and lens/cornea genes (nexus format); (2) maximum likelihood (RAxML) gene trees with bootstrap support values; (3) maximum-likelihood species trees with bootstrap values based on concatenated alignments; (4) alignments for codeml analyses (frameshift insertions removed, stop codons replaced with Ns); (5) species trees for codeml analyses; (6) example codeml control files; (7) coverage for validated autapomorphic inactivating mutations in *Chrysochloris asiatica* and the DNA Zoo individual of *Notoryctes typhlops* (Table S1); (8) inactivating mutations in taxa other than chrysochlorids and *N. typhlops* (Table S2); (9) an Excel calculator that was used for calculating inactivation times of eye-specific genes (Table S3); and (10) splice site mutation (GT to AT) in intron 6 of the *PDE6B* gene in one of the individuals of *N. typhlops* (Figure S1). New WGS Illumina data for *Amblysomus hottentotus* and *Notoryctes typhlops* are available at NCBI BioProject PRJNA1010772.
