# Supplementary material for: Three Blind Moles: Molecular Evolutionary Insights on the Tempo and Mode of Convergent Eye Degeneration in Notoryctes typhlops (Southern Marsupial Mole) and Two Chrysochlorids (Golden Moles)
Source: Genes (Basel). 2023 Oct 28;14(11):2018. doi: 10.3390/genes14112018 (PMC10671557; doi:10.3390/genes14112018)
Supplement: Supplementary file 1 [file genes-14-02018-s001.zip › Supplementary Tables and Figures/Table S2 (Inactivating mutations in other taxa).pdf]

Table S2. Inactivating mutations in eye-specific genes in taxa other than Chrysochloridae and *Notoryctes typhlops*. Numerical positions refer to coding sequence (CDS) alignments.

Gene	Inactivating Mutations				
	Strigocuscus gymnotis	*Myrmecobius fasciatus*	Tenrecidae	*Echinops telfairi*	*Microgale talazaci*
OPN1SW	88-90S (E1), 955-965D (E5)				
GRK1		1351-1353S (E6)			
SLC24A1		2341D (E4)			
GRK7				265D (E1), 320D (E1), 546-547D (E1), In1Do (TT), 1036D (E2), 1138-1157D (E3), 1477I (E4)	
CRYBA4			No BLAST results		
CRYBB1			In4Do (AT)	1-3SCM (E1), 10D (E1), 93D (E1), 136-140D (E1), 208-210S (E2), 322-324S (E3), 360-379D (E3), 391I (E4), I3E4BD (4 bp of intron and two bp of exon [428-429]), 518-520S (E4), 600-602S (E5), 612-614S (E5), 654-656S (E5), 697-699S (E5)	117D (E1), 175-293NBR (E2), 364-366S (E3), 413-415S (E3), 448D (E4),550-553I (E4), 558-560S (E4), 627-629S (E5), 654-656S (E5), 690I (E5)
CRYBB3				In4Do (GG), 317-319S (E3), 418-420S (E4), 596-598S (E5), 609D (E5)	No BLAST results

Abbreviations: Ac, acceptor splice site; AfSI, AfroSINE insertion; BD, boundary deletion; D, deletion; Do, donor splice site; E, exon; I, insertion; In, intron; INV, inversion; NBR = no BLAST results and possible deletion of exon(s) or gene; NRM = no reads mapped and possible deletion of exon(s) or gene; S, premature stop codon, SCM, start codon mutation, TCM, termination codon mutation; UTR, untranslated region.

*Locations of inactivating mutations in exons are based on alignments for complete protein-coding sequences including frameshift insertions when present.
