# Supplementary figures and images for: Three Blind Moles: Molecular Evolutionary Insights on the Tempo and Mode of Convergent Eye Degeneration in Notoryctes typhlops (Southern Marsupial Mole) and Two Chrysochlorids (Golden Moles)
Source: Genes (Basel). 2023 Oct 28;14(11):2018. doi: 10.3390/genes14112018 (PMC10671557; doi:10.3390/genes14112018)

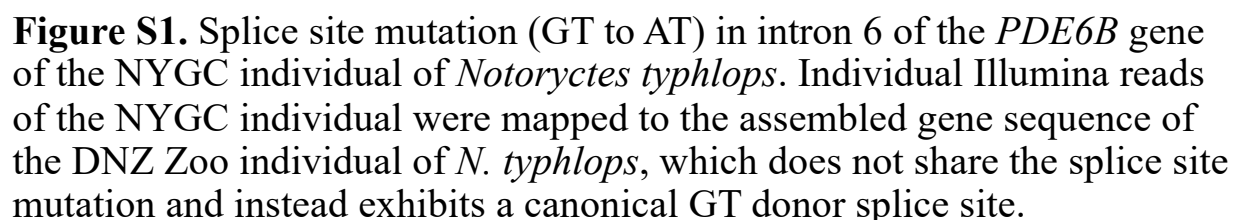

Supplement: Supplementary file 1 [file genes-14-02018-s001.zip › Supplementary Tables and Figures/Figure S1.pdf]
